# Glucose Homeostatic Law: Insulin Clearance Predicts the Progression of Glucose Intolerance in Humans

**DOI:** 10.1371/journal.pone.0143880

**Published:** 2015-12-01

**Authors:** Kaoru Ohashi, Hisako Komada, Shinsuke Uda, Hiroyuki Kubota, Toshinao Iwaki, Hiroki Fukuzawa, Yasunori Komori, Masashi Fujii, Yu Toyoshima, Kazuhiko Sakaguchi, Wataru Ogawa, Shinya Kuroda

**Affiliations:** 1 Department of Biological Sciences, Graduate School of Science, University of Tokyo, Bunkyo-ku, Tokyo, Japan; 2 Division of Diabetes and Endocrinology, Department of Internal Medicine, Kobe University Graduate School of Medicine, Chuo-ku, Kobe, Japan; 3 Department of Computational Biology, Graduate School of Frontier Sciences, University of Tokyo, Bunkyo-ku, Tokyo, Japan; 4 Faculty of Medicine, University of Tokyo, Bunkyo-ku, Tokyo, Japan; RIKEN Center for Integrative Medical Sciences, JAPAN

## Abstract

Homeostatic control of blood glucose is regulated by a complex feedback loop between glucose and insulin, of which failure leads to diabetes mellitus. However, physiological and pathological nature of the feedback loop is not fully understood. We made a mathematical model of the feedback loop between glucose and insulin using time course of blood glucose and insulin during consecutive hyperglycemic and hyperinsulinemic-euglycemic clamps in 113 subjects with variety of glucose tolerance including normal glucose tolerance (NGT), impaired glucose tolerance (IGT) and type 2 diabetes mellitus (T2DM). We analyzed the correlation of the parameters in the model with the progression of glucose intolerance and the conserved relationship between parameters. The model parameters of insulin sensitivity and insulin secretion significantly declined from NGT to IGT, and from IGT to T2DM, respectively, consistent with previous clinical observations. Importantly, insulin clearance, an insulin degradation rate, significantly declined from NGT, IGT to T2DM along the progression of glucose intolerance in the mathematical model. Insulin clearance was positively correlated with a product of insulin sensitivity and secretion assessed by the clamp analysis or determined with the mathematical model. Insulin clearance was correlated negatively with postprandial glucose at 2h after oral glucose tolerance test. We also inferred a square-law between the rate constant of insulin clearance and a product of rate constants of insulin sensitivity and secretion in the model, which is also conserved among NGT, IGT and T2DM subjects. Insulin clearance shows a conserved relationship with the capacity of glucose disposal among the NGT, IGT and T2DM subjects. The decrease of insulin clearance predicts the progression of glucose intolerance.

## Introduction

A feedback loop linking insulin and glucose plays an essential role in glucose homeostasis [1]. This feedback loop, in which an increase in the blood glucose level stimulates insulin secretion and an increase in blood insulin concentration lowers the blood glucose level, has been analyzed with regard to two major aspects: insulin secretion and insulin sensitivity [2]. A number of clinical indices that reflect these two aspects have been proposed and shown to be of use for analysis of glucose homeostasis and the pathology of glucose intolerance [3]. Given that these indices are usually determined by blood parameters under specific conditions [3], however, it is unclear whether they reflect all aspects of insulin secretion and insulin sensitivity.

Application of a mathematical model is one approach to overcome the limitation of actual measurement of clinical parameters [4–18]. The minimal model, derived from time series data of a frequently sampled intravenous glucose tolerance test (FSIVGTT), is an example of a successful mathematical model for assessment of glucose homeostasis [19, 20]. Mathematical models based on time series data of an oral glucose tolerance test (OGTT) have also been developed and shown to be of clinical utility [21–30]. However, given that blood glucose and insulin levels during an FSIVGTT or OGTT are mutually influenced via the negative feedback loop, it is difficult to accurately determine parameters for insulin sensitivity and insulin secretion, and a key feature of the negative feedback loop may therefore remain to be uncovered.

An ideal approach to the study of biological phenomena regulated by a feedback loop is to analyze time series data obtained after severance of the feedback relation by clamping one of the components. In this study, to further understand the regulation of glucose homeostasis. We thus generated mathematical model with time series data of consecutive hyperglycemic and hyperinsulinemic-euglycemic clamps, in which indices for insulin secretion and insulin sensitivity are independently evaluated without an effect of the feedback relation. We have analyzed parameters of the model to uncover previously unrecognized relation in factors that regulate the capacity of glucose tolerance

## Results

### Mathematical model based on time series data of consecutive hyperglycemic and hyperinsulinemic-euglycemic clamps

**Fig 1:**
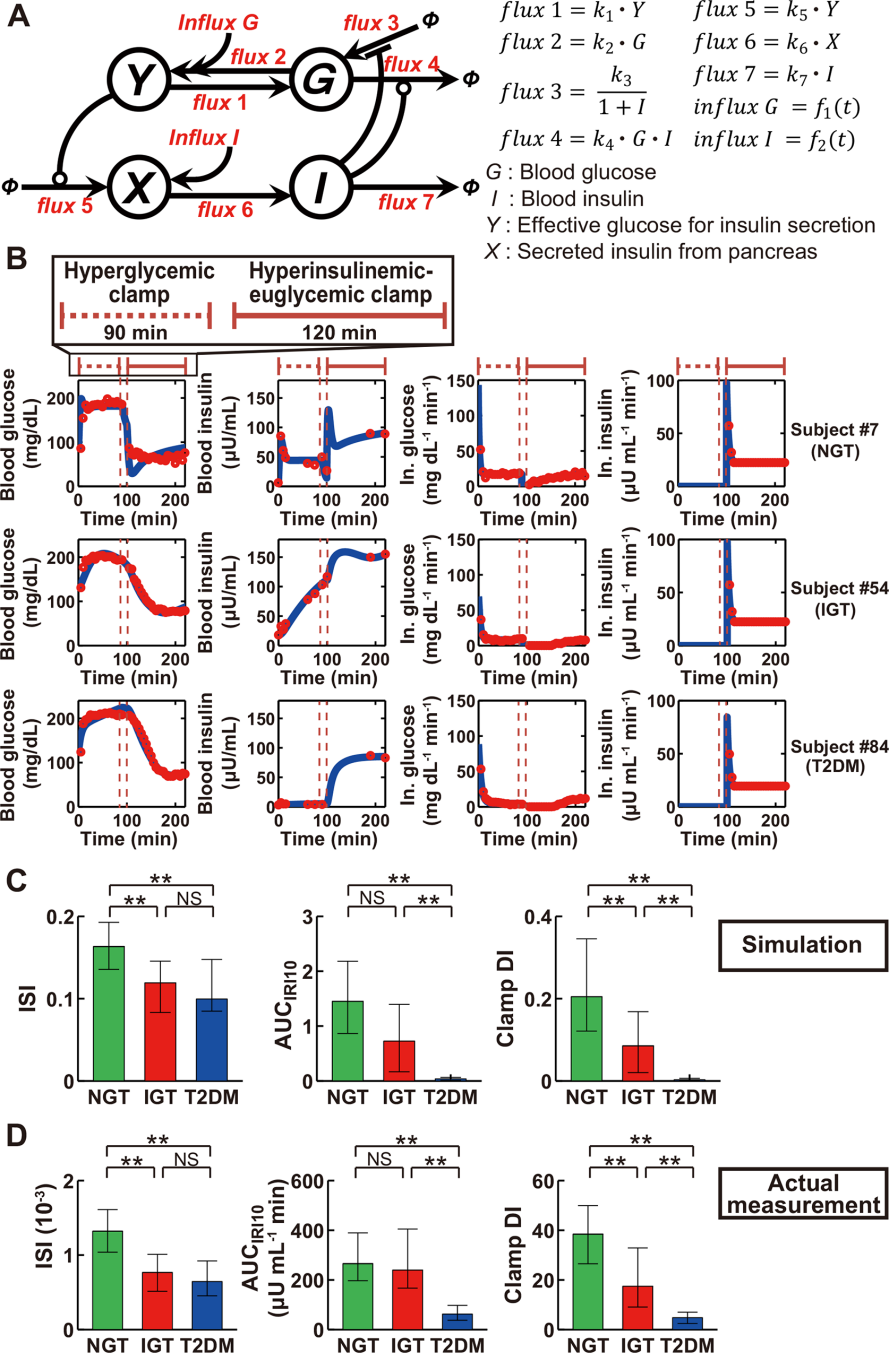
Feedback model represents essential features of NGT, IGT, and T2DM subjects. (A) The structure of the feedback model. Arrows indicate fluxes, and lines ending in circles or bars indicate activation and inactivation, respectively. Φ denotes a fixed value. Functions *f*_1_(*t*) and *f*_2_(*t*) are described in Materials and Methods. (B) The time courses of blood glucose, blood insulin, infused (In.) glucose, and infused insulin for typical NGT, IGT, and T2DM subjects. Red circles and blue curves are actual measurement data and simulation results, respectively. The simulated values of each variable were rescaled to absolute concentration and plotted. Periods of hyperglycemic and hyperinsulinemic-euglycemic clamps are indicated at the top. (C, D) The insulin sensitivity index (ISI), insulin secretion as the incremental area under the curve of immunoreactive insulin concentration during the first 10 min (AUC_IRI10_) of the hyperglycemic clamp, and the clamp disposition index (DI), which is given by the product of ISI and AUC_IRI10_, both for the simulation (C) and actual measurements (D). Note that the indices in the simulation are calculated by normalized time course, and are dimensionless value. *P < 0.05, **P < 0.01 by the Steel-Dwass test. NS, not significant.

We developed a mathematical model of the feedback loop that links glucose and insulin with the use of time series data of blood glucose and insulin concentrations during consecutive hyperglycemic and hyperinsulinemic-euglycemic clamps (Fig 1A). In this model, the variables *G* and *I* represent blood glucose and insulin concentrations, respectively. The variable *Y* is affected by *G* and corresponds to the effective glucose concentration on induction of variable *X*, which can be regarded as insulin secreted from pancreatic β-cells. *G* regulates *I* through *Y* and *X*, and *I* directly regulates *G*, constituting the feedback loop.

Fluxes in the model indicate the flux between variables. Fluxes that regulate insulin secretion are *flux* 5, *flux* 6, and *flux* 7, where *flux* 5 depends on *Y* and corresponds to insulin secretion in response to an effective glucose concentration, and whose parameter *k*_5_ corresponds to the rate constant of insulin secretion; *flux* 6 depends on *X* and corresponds to systemic circulation of insulin from the pancreas to target organs; and *flux* 7 depends on *I* and corresponds to insulin clearance, and whose parameter *k*_7_ corresponds to the rate constant of insulin clearance. Fluxes that regulate insulin sensitivity are *flux* 3 and *flux* 4, where *flux* 3 is inhibited by *I* and corresponds to glucose production by target organs, and *flux* 4 depends on both *G* and *I* and corresponds to glucose uptake that is facilitated by both glucose and insulin. Systemic circulation of glucose from the pancreas or of infused glucose to target organs is represented by *flux* 1, whereas *flux* 2 corresponds to circulation of glucose to the pancreas. *Influx G* and *influx I* correspond to infused glucose and insulin during the consecutive hyperglycemic and hyperinsulinemic-euglycemic clamps, respectively.

We estimated parameters of the model for each of 113 subjects (47 NGT, 16 IGT, and 50 T2DM subjects) (S1 Table) using normalized data of the consecutive hyperglycemic and hyperinsulinemic-euglycemic clamps. We simulated the time course of blood glucose and insulin concentrations in the clamp analyses, with one example each of NGT, IGT, and T2DM subjects being shown in Fig 1B and the results for all subjects in S1 Fig.

To confirm that the simulation appropriately reflects characteristics of the subjects, we evaluated the consistency between the clinical indices calculated from the actual measurements and those calculated from the simulation data. ISI calculated from the time course of blood glucose and insulin concentrations in the simulation was greater for NGT subjects than for IGT or T2DM subjects, whereas it did not differ significantly between the latter two groups of subjects (Fig 1C). AUC_IRI10_ calculated in the simulation was similar in NGT and IGT subjects and significantly greater in these two groups than in T2DM subjects. These characteristics of ISI and AUC_IRI10_ in the simulation mimicked those calculated from the actual measurements (Fig 1D). The disposition index (DI), originally defined as the product of indices for insulin secretion and insulin sensitivity determined by the minimal model [19], is thought to reflect the capacity for insulin secretion adjusted for insulin sensitivity and therefore to represent the integrated capacity for glucose disposal. An analog of DI determined by consecutive glucose clamps (clamp DI) has been shown to possess clinical characteristics similar to those of the original DI [31]. A simulated clamp DI, the product of simulated ISI and simulated AUC_IRI10_, decreased significantly with progression from NGT to IGT to T2DM (Fig 1C). This characteristic was also similarly observed with clamp DI calculated from the actual measurements (Fig 1D). These results thus suggested that the simulation model retains well the essential characteristics of the capacity for glucose disposal among the study subjects.

### Parameters in the model characterize progression of glucose intolerance

**Fig 2:**
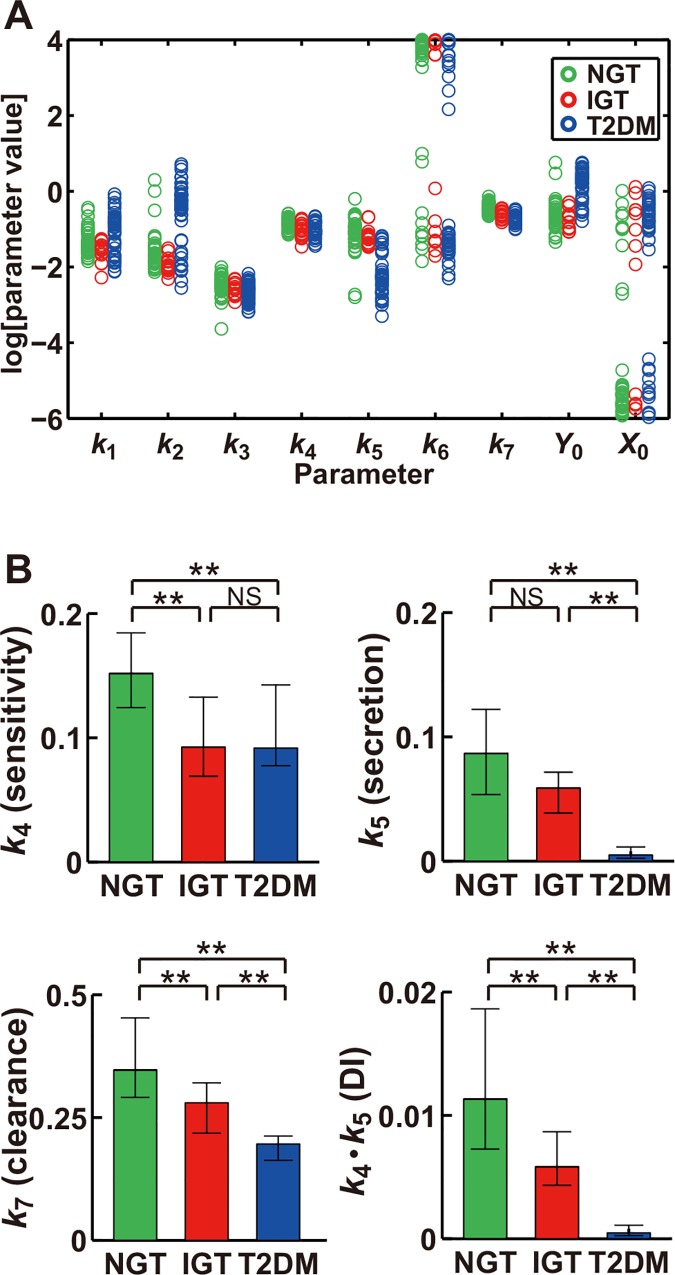
Parameters in the feedback model characterize glucose tolerance in NGT, IGT, and T2DM subjects. (A) The estimated parameters for NGT (green), IGT (red), and T2DM (blue) subjects. (B) The parameters for NGT, IGT, and T2DM subjects, with *k*_4_, *k*_5_, *k*_7_, and *k*_4_∙*k*_5_ being rate constants of insulin sensitivity, insulin secretion, insulin clearance, and DI, respectively. *P < 0.05, **P < 0.01 (Steel-Dwass test).

To identify parameters that characterize the progression of glucose intolerance, we investigated specific parameters of the model that differed significantly among the NGT, IGT, and T2DM groups. We plotted the estimated parameter distributions in the three groups of subjects and subjected them to statistical analysis with the nonparametric Steel-Dwass test [32] (Fig 2). The parameters *Y*_0_ and *k*_5_ differed significantly between T2DM and the other two groups; *k*_4_ differed significantly between NGT and the other two groups; and *k*_2_ and *k*_7_ differed significantly among all three groups of subjects.

*Y*_0_ is the initial effective blood glucose concentration for insulin secretion (Fig 1A), which corresponds to the fasting plasma glucose (FPG) concentration in the actual measurements. It is thus reasonable that *Y*_0_ is greater in T2DM subjects than in the other two groups. The parameter *k*_4_ is the rate constant for glucose uptake facilitated by insulin and glucose (Fig 1A), which conceptually corresponds to insulin sensitivity, and the result that *k*_4_ for NGT subjects was significantly higher than that for IGT or T2DM groups is consistent with the finding that both simulated and actual ISI for IGT or T2DM groups was lower than that for NGT (Fig 1C and 1D). The parameter *k*_5_, the rate constant for insulin secretion, conceptually corresponds to the capacity for insulin secretion. The finding that *k*_5_ for the T2DM group was significantly smaller than that for the NGT or IGT groups thus indicated that insulin secretion is reduced in T2DM subjects compared with NGT and IGT subjects, consistent with the result that both simulated and actual AUC_IRI10_ values for T2DM were smaller than those for the other two groups (Fig 1C and 1D).

We next performed parameter sensitivity analysis for ISI and AUC_IRI10_ in the simulation (S3 Table) in order to evaluate the sensitivity of each parameter for reproduction of the simulated time courses of blood glucose and insulin concentrations. We examined all 70 parameters including the seven rate constants (*k*_1_ to *k*_7_), the 21 products of each pair of rate constants, and the 42 quotients for each two rate constants. This analysis revealed that the most sensitive parameter for ISI was *k*_4_, and that for AUC_IRI10_ was *k*_7_, although *k*_5_ was also one of the most sensitive parameters for AUC_IRI10_ (S3 Table). These results are consistent with the functional roles of *k*_4_ (insulin sensitivity) and *k*_5_ (insulin secretion) in the feedback model.

The product of *k*_4_ and *k*_5_ (*k*_4_∙*k*_5_), which conceptually corresponds to DI, decreased significantly with progression from NGT to IGT to T2DM (Fig 2B), as did the simulated and actual DIs (Fig 1C and 1D). Of note, the parameter *k*_7_, the rate constant of insulin clearance, also significantly declined with such progression (Fig 2B). These results thus suggested that *k*_7_ is correlated with *k*_4_∙*k*_5_, with DI, and, consequently, with the capacity for glucose disposal.

Although *k*_2_, the rate constant for flux from glucose to effective glucose for insulin secretion, differed significantly among the three groups, it decreased in the rank order T2DM > NGT > IGT, indicating that *k*_2_ does not represent progression of glucose intolerance. The physiological relevance of this difference remains to be elucidated.

### Parameters that characterize the ability of glucose disposal

**Fig 3:**
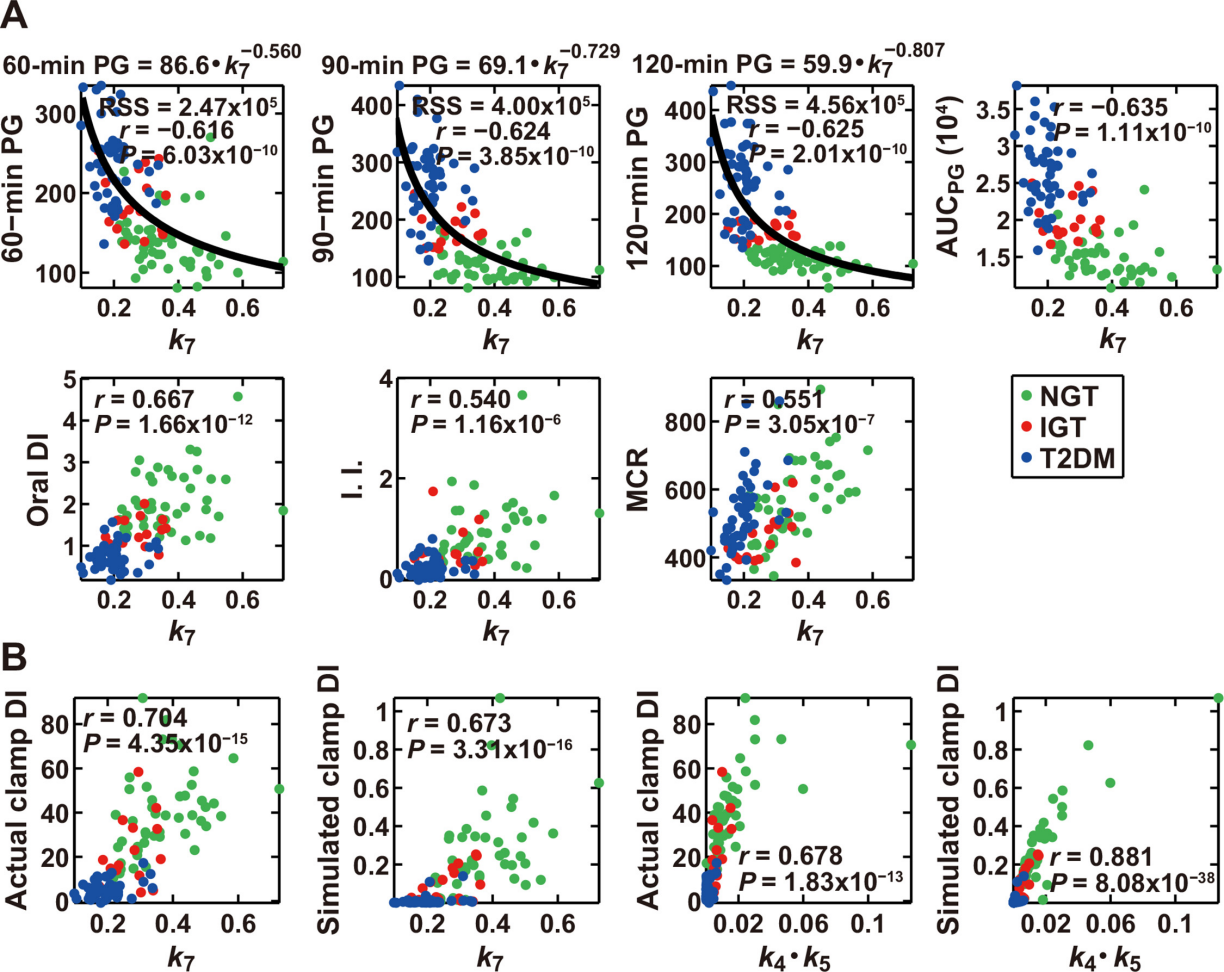
Model parameters that characterize clinical indices of glucose tolerance. (A) The scatter plots for *k*_7_ in the model versus actual measured clinical indices. Each circle corresponds to the values for a single subject. Green, red and blue indicate NGT, IGT, and T2DM subjects, respectively; *r* is the correlation coefficient; and *P* values are for testing the hypothesis of no correlation. The distribution of *k*_7_ and plasma glucose concentration (PG) at individual time points during the OGTT was fitted by a power function (see Materials and Methods), which is also indicated at the top of the corresponding plots. RSS, residual sum of the square between the parameter and the fitted curve. I.I., insulinogenic index; MCR, metabolic clearance rate. (B) The scatter plots for the indicated parameters in the model versus clinical indices for both actual measurements and the simulation, as indicated. The units of the indicated indices are shown in Materials and Methods.

Given that our results suggested that the rate constant for insulin clearance, *k*_7_, might be related to the capacity for glucose disposal, we next examined the correlation between parameters of the mathematical model and various clinical indices determined by the OGTT performed in each subject (S4 Table and S2 Fig). Among the 70 parameters consisting of each rate constant as well as the products and quotients for each pair of rate constants, *k*_7_ showed the highest correlation with 90-min plasma glucose (PG) and AUC_PG_ values, the latter being the integrated glucose concentration during the OGTT, as well as the second highest correlation with FPG, 60-min PG, and 120-min PG during the OGTT (S4 Table and Fig 3A), indicating that *k*_7_ is highly correlated with the capacity for glucose disposal. An analog of DI calculated from parameters measured during an OGTT (oral DI) has been proposed and shown to possess characteristics similar to those of the original DI (Materials and Methods). We found that *k*_7_ also exhibited the highest correlation with oral DI (Fig 3A), whereas the correlation of *k*_7_ with each index of insulin sensitivity or insulin secretion, including the components of oral DI (Matsuda index and AUC_IRI120/PG120_), was not especially high (S4 Table). The tight relation between the capacity for glucose disposal and *k*_7_ was thus confirmed by the results of the OGTT. In addition, *k*_7_ showed the highest correlation with the insulinogenic index (Fig 3A), which is thought to be an important determinant of the capacity for glucose disposal.

The parameter that exhibited the highest correlation with MCR (metabolic clearance rate) calculated from the insulin infusion rate and circulating insulin levels during hyperinsulinemic-euglycemic clamp analysis [33], was *k*_7_ (S4 Table and Fig 3A). MCR is a clinical index of insulin clearance rate, and corresponds to *k*_7_ in the mathematical model. However, MCR was not highly correlated with either 30-min PG (*r* = –0.22), 60-min PG (*r* = –0.22), 90-min PG (*r* = –0.16), 120-min PG (*r* = –0.17), AUC_PG_ (*r* = –0.20), oral DI (*r* = 0.23), or clamp DI (*r* = 0.32) (S5 Table and S3 Fig). It should be noted that MCR is not exactly the same as insulin clearance; MCR indicates the ratio between insulin infusion rate and blood insulin level at steady state whereas *k*_7_ indicates the rate of insulin degradation, which is denoted as insulin clearance in this study.

### Conserved relationship between insulin clearance and clinical indices

**Fig 4:**
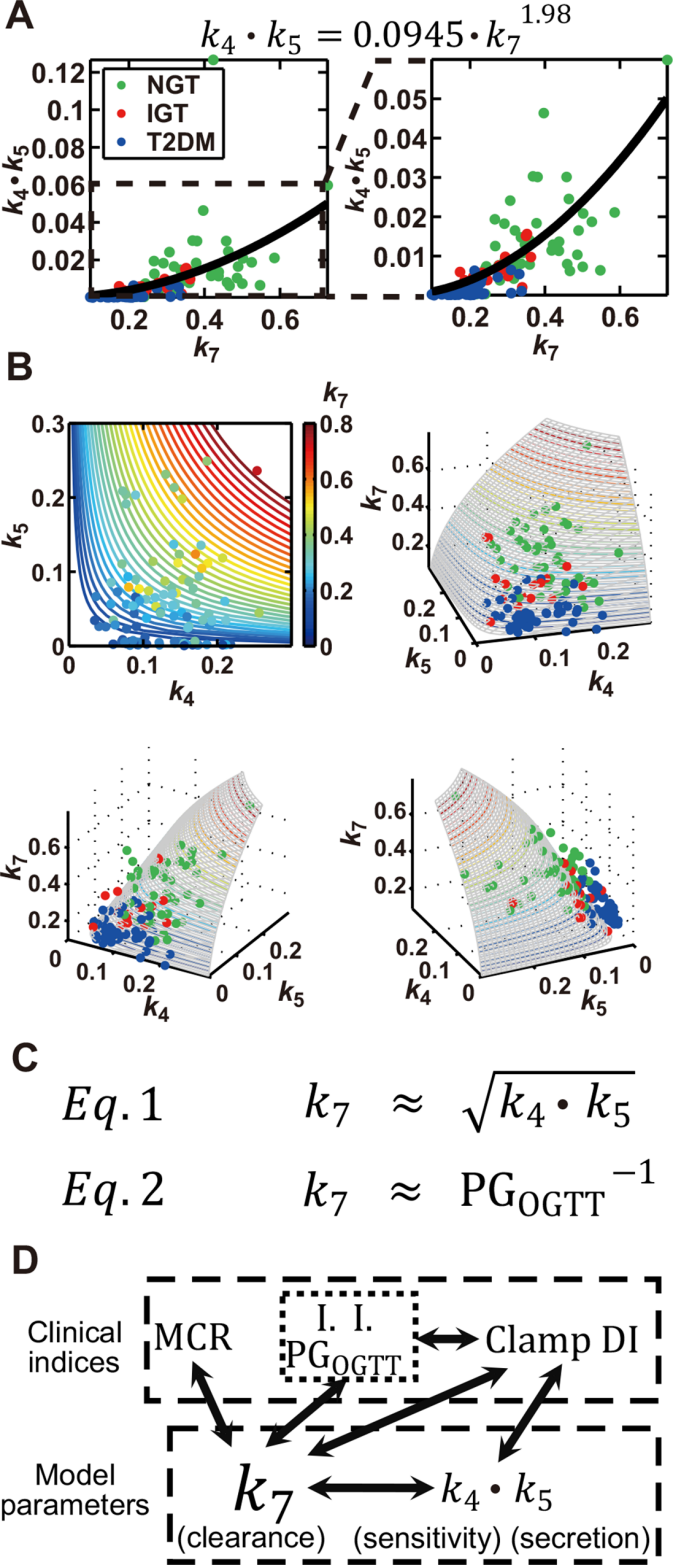
Square-law relation between rate constants of insulin clearance (*k*_7_) and DI (*k*_4_∙*k*_5_). (A) The scatter plot of *k*_7_ and *k*_4_∙*k*_5_, with circles indicating the parameters of each subject and the curve fitted with the estimated power function shown at the top. The boxed region in the left plot is expanded in the plot on the right. (B) The contour plot of *k*_4_, *k*_5_, and *k*_7_ (upper left), with the value of *k*_7_ being indicated by colors, as well as three-dimensional plots of *k*_4_, *k*_5_, and *k*_7_, where circle colors of green, red and blue indicate NGT, IGT, and T2DM subjects, respectively. RSS was 0.0168. Parameters with *k*_4_ ≤ 0.3 and *k*_5_ ≤ 0.3 are plotted. (C) The square-law relation (*Eq* 1) and the inverse proportion relation (*Eq* 2) inferred from the feedback model. PG_OGTT_ indicates postprandial plasma glucose level at each time point during the OGTT. (D) The relation between clinical indices and parameters in the model. Solid arrows indicate the relations for which |*r*| ≥ 0.5.

The clinical indices that differed significantly among each of the NGT, IGT, and T2DM groups were actual and simulated clamp DI (Fig 1C and 1D), and the parameters in the model that differed significantly among each of the three groups were *k*_7_ and *k*_4_∙*k*_5_ (Fig 2B). We therefore examined the correlation between clamp DI and these model parameters (Fig 3B). The actual and simulated clamp DI showed a high correlation with both *k*_7_ and *k*_4_∙*k*_5_. The similarity between *k*_7_ and *k*_4_∙*k*_5_ suggests the existence of an unrecognized relation between insulin clearance and both insulin sensitivity and insulin secretion. We further investigated the relation between *k*_4_∙*k*_5_ and *k*_7_ by fitting their distribution with a power function, with the power index appearing to be 1.98 (Fig 4A), indicative of a square-law relation between *k*_7_ and *k*_4_∙*k*_5_ (*Eq* 1 in Fig 4C). We plotted the distribution of *k*_4_, *k*_5_, and *k*_7_ in a contour plot (Fig 4B), with each parameter appearing along the curve surface of the square-law equation. Analysis of our mathematical model thus allowed us to infer the hidden square-law relation between two functionally different parameters, *k*_4_∙*k*_5_ and *k*_7_.

## Discussion

In this study, we estimated parameters from the time courses of consecutive hyperglycemic and hyperinsulinemic-euglycemic clamps and inferred a square-law relation between the rate constant of insulin clearance (*k*_7_) and the product of the rate constants of insulin sensitivity (*k*_4_) and insulin secretion (*k*_5_) (*Eq* 1 in Fig 4C). Of note, the right and left sides of the fitted equation in Fig 4A have the same dimension, and the ratio between *k*_7_ and $\sqrt{k_{4}\cdot k_{5}}$ always remains constant among NGT, IGT, and T2DM subjects. The ratio between *k*_7_ and $\sqrt{k_{4}\cdot k_{5}}$ may therefore indicate a homeostasis constant of glucose tolerance that constrains the rate constant of insulin clearance as well as those of insulin sensitivity and insulin secretion. As far as we are aware, such a relation between insulin secretion, sensitivity, and clearance has not previously been proposed on the basis of actual measurement of clinical indices. The reason why we have discovered this relation may be because of using both consecutive hyperglycemic and hyperinsulinemic-euglycemic clamps and mathematical modeling. Hyperglycemic clamp data involves quantitative relation between insulin clearance and secretion, and hyperinsulinemic-euglycemic clamp data involves quantitative relation between insulin clearance and sensitivity. In addition, because model structure constrained the relation between insulin clearance, secretion and sensitivity, parameter estimation based on consecutive hyperglycemic and hyperinsulinemic-euglycemic clamps uncovered quantitative relation between *k*_7_ and $\sqrt{k_{4}\cdot k_{5}}$ conserved among NGT, IGT, and T2DM subjects. Our study has thus elucidated an unrecognized relation between factors that play important roles in the regulation glucose homeostasis.

The role of insulin clearance in glucose intolerance has thus far been studied, however, the role of insulin clearance remains controversial. Insulin clearance has been reported to be negatively correlated with the progression of glucose intolerance [34–40]. On the other hand, it has been reported that insulin clearance is increased in subjects carrying diabetes-susceptible gene [41], and that insulin clearance is not affected by decreasing insulin secretion by free fatty acid administration [42]. The controversial role of insulin clearance may be because the data obtained by OGTT or single clamp alone contain less information regarding insulin clearance than that obtained by the consecutive clamps in this study, or because the insulin clearance directly calculated from the measured data is less quantitative than that obtained by the mathematical modeling. Our study strongly supports the conclusion in the former studies [34–40].

Insulin secretion and insulin sensitivity are thought to determine the capacity for glucose disposal, and DI, defined as the product of indices for these two parameters, has indeed been shown to reflect the integrated capacity for glucose disposal. Given that *k*_4_∙*k*_5_ conceptually corresponds to DI, it is not surprising that it correlates with parameters for glucose disposal to a certain extent. An unexpected finding in the current study, however, is that *k*_*7*_, the rate constant of insulin clearance, showed the highest or one of the highest correlations with PG level as well as with AUC_PG_ during an OGTT (*Eq* 2 in Fig 4C). This finding, together with the high correlations of *k*_7_ with oral and clamp DIs, is indicative of the strong relation between insulin clearance and the capacity for glucose disposal.

Although our analysis suggests the existence of a previously unrecognized relation among the three important components of insulin-regulated glucose homeostasis—insulin secretion, insulin sensitivity, and insulin clearance—the mechanism for the overall control of these components remains unknown. Insulin is degraded in cells after its receptor-mediated internalization, with this internalization being tightly linked to the intrinsic tyrosine kinase activity of the insulin receptor, a key signaling function of this protein [43]. Both insulin clearance and insulin sensitivity may thus be strongly influenced by the integral of the abundance and activity of the insulin receptor. Indeed, insulin clearance in the liver has been shown to be an important determinant of insulin sensitivity in dogs [44]. Insulin secretion in the living body manifests a pulsatile pattern, and the maintenance of this pattern is thought to be important for both insulin sensitivity and glucose tolerance. Although the mechanistic link between pulsatile insulin secretion and insulin sensitivity is not fully understood, we have recently shown that certain elements of insulin signaling respond not to the absolute value but to the temporal profile of insulin concentration [45, 46]. Loss of the pulsatile pattern of insulin secretion induced by partial pancreatectomy was also found to result in a decrease not only in insulin sensitivity but also in insulin clearance [47]. Theoretically, if insulin clearance is impaired, the retention period of circulating insulin would be expected to be prolonged and rapid temporal changes in insulin secretion would not be well reflected by circulating insulin levels [48], suggesting that the pulsatile pattern is observed only when insulin clearance is fully operative. We found that *k*_7_ also showed the highest correlation with the insulinogenic index, an index of early insulin secretion. It is possible that the higher *k*_*7*_ is, the more the rapid temporal changes in insulin secretion are reflected in circulating insulin levels. A high level of insulin clearance is thus likely important for full transmission of information from pancreatic β-cells to the whole body. This notion may be related, at least in part, to the underlying relation between the secretion, sensitivity, and clearance of insulin.

Many mathematical models of systemic insulin-glucose dynamics have been reported [5–20, 23, 25]. One of the differences between these previous models and the present model is incorporation of the infusion of glucose and insulin in our model. Another key difference is that we performed both hyperglycemic and hyperinsulinemic-euglycemic clamps, which allowed us to evaluate indices for insulin secretion and insulin sensitivity without an effect of the feedback relation. To examine whether both hyperglycemic and hyperinsulinemic-euglycemic clamps are necessary to uncover the relation between insulin clearance and glucose intolerance, we estimated parameters using hyperglycemic or hyperinsulinemic-euglycemic clamp data alone, and found that insulin clearance did not significantly differ between three groups (S4 Fig). In the model with hyperinsulinemic-euglycemic clamp, insulin clearance declined from NGT to IGT, suggesting that addition of hyperglycemic clamp data is necessary to find the difference of insulin clearance from IGT to T2DM.

In conclusion, our mathematical analysis has revealed that the rate constant of insulin clearance represents both the capacity for glucose disposal and a product of insulin sensitivity and insulin secretion among subjects with a range of glucose tolerance (Fig 4D). Further elucidation of underlying mechanism of this law may provide important insight into the physiology of glucose homeostasis and the pathology of glucose intolerance.

## Materials and Methods

### Subjects and measurements

Study subjects were recruited as described previously [31] at Kobe University Hospital from October 2008 to June 2014. The study was approved by the ethics committee of Kobe University Hospital, and written informed consent was obtained from all subjects. Consecutive hyperglycemic and hyperinsulinemic-euglycemic clamp analyses as well as a standard 75-g OGTT were performed within a period of 10 days as described previously [31]. In brief, before the onset of the consecutive clamp analyses, fasting plasma glucose and serum insulin concentrations were measured as the data for time zero. From 0 to 90 min, a hyperglycemic clamp was performed by intravenous infusion of a bolus of glucose (9622 mg/m^2^) within 15 min followed by that of a variable amount of glucose to maintain the plasma glucose level at 200 mg/dL. Ten minutes after the end of the hyperglycemic clamp, a 120-min hyperinsulinemic-euglycemic clamp was initiated by intravenous infusion of human regular insulin (Humulin R, Eli Lilly Japan K.K.) at a rate of 40 mU m^–2^ min^–1^ and with a target plasma glucose level of 90 mg/dL. For NGT and IGT subjects whose plasma glucose levels were <90 mg/dL, the plasma glucose concentration was clamped at the fasting level. We measured the plasma glucose level every 1 min during the clamp analyses and obtained the 5-min average values. We also measured the insulin level in serum samples collected at 5, 10, 15, 60, 75, 90, 100, 190, and 220 min after the onset of the tests. First-phase insulin secretion during the hyperglycemic clamp was defined as the incremental area under the immunoreactive insulin (IRI) concentration curve (μU mL^–1^ min) from 0 to 10 min (AUC_IRI10_). An index of insulin sensitivity derived from the hyperinsulinemic-euglycemic clamp, the insulin sensitivity index (ISI), was calculated by dividing the mean glucose infusion rate during the final 30 min of the clamp (mg kg^–1^ min^–1^) by both the plasma glucose (mg/dL) and serum insulin (μU/mL) levels at the end of the clamp and then multiplying the resulting value by 100. A clamp-based analog of the disposition index, which we termed the clamp disposition index (clamp DI), was calculated as the product of AUC_IRI10_ and ISI, as described previously [31]. The metabolic clearance rate (MCR) [33], an index of insulin clearance, was calculated by dividing the insulin infusion rate at the steady state (1.46 mU kg^–1^ min^–1^) by the increase in the insulin concentration above the basal level in the hyperinsulinemic-euglycemic clamp as described previously [31]: [1.46 (mU kg^–1^ min^–1^) × body weight (kg) × body surface area (m^2^)/(end IRI–fasting IRI) (μU/mL)], where body surface area is defined as [(body weight (kg))^1/2^ × (body height (cm))^1/2^ / 60] (Mosteller formula). Calculation of other clinical indices is described in the following section (Calculation of Clinical Indices). Fifty NGT, 18 IGT, and 53 T2DM subjects (121 in total) were initially used for parameter estimation. Three NGT, two IGT, and three T2DM subjects were subsequently eliminated as outliers (see Determination of parameter outliers below), and the remaining 47 NGT, 16 IGT, and 50 T2DM subjects were studied (S1 Table). The actual data for all 121 subjects are shown in S6 Table.

### Mathematical model

We constructed the feedback model (Fig 1A) as follows:

$$\begin{array}{l} \frac{dG}{dt}=flux\;1-flux\;2+flux\;3-flux\;4=k_{1}\cdot Y-k_{2}\cdot G+\frac{k_{3}}{1+I}-k_{4}\cdot G\cdot I\\ \frac{dI}{dt}=flux\;6-flux\;7\;=k_{6}\cdot X-k_{7}\cdot I\\ \frac{dY}{dt}=-flux\;1+flux\;2+influx\;G\;=-k_{1}\cdot Y+k_{2}\cdot G+f_{1}(t)\\ \frac{dX}{dt}=flux\;5-flux\;6+influx\;I\;=k_{5}\cdot Y-k_{6}\cdot X+f_{2}(t) \end{array}$$

where the variables *G* and *I* denote normalized blood glucose and insulin concentrations, respectively (see Parameter estimation below), and the model parameters for each subject are estimated to reproduce the time course of the corresponding clamp data. The actual glucose infusion rate (*GIR* [mg kg^–1^ min^–1^]) and insulin infusion rate (*IIR* [mU kg^–1^ min^–1^]) were converted to the corresponding blood concentrations (*cGIR* and *cIIR*, respectively) as follows:

$$\begin{array}{l} cGIR({\text{mg dL}}^{-1}\;{\text{min}}^{-1})=\frac{GIR({\text{mg kg}}^{-1}\;{\text{min}}^{-1})\cdot BW}{BW\cdot BV}\cdot 100\\ cIIR(\mu \;\text{U}\;{\text{mL}}^{-1}\;{\text{min}}^{-1})=\frac{IIR({\text{mU kg}}^{-1}\;{\text{min}}^{-1})\cdot BW}{BW\cdot BV}\cdot 1000 \end{array}$$

where *BW* and *BV* denote body weight and blood volume (75 and 65 mL/kg for men and women, respectively [49]), respectively.

In the model, glucose and insulin infusions are represented by *influx G* and *influx I*, respectively. These fluxes follow the nonlinear functions *f*_1_ and *f*_2_ that predict glucose and insulin infusion concentrations, respectively. Given that the infusion protocol differed between hyperglycemic (from 0 to 90 min) and hyperinsulinemic-euglycemic (from 100 to 220 min) clamps, the functions of the infusion rates also differ between the hyperglycemic clamp (*f* ^0–90^) and the hyperinsulinemic-euglycemic clamp (*f* ^100–220^) and are given by the following equations:

$$f_{1}^{0-90}(t)=gc_{1}\cdot \text{exp}(gc_{2}\cdot t)+gc_{3},\;f_{2}^{0-90}(t)=0$$

$$f_{1}^{100-220}(t)=gi_{1}\cdot \frac{{\left((t-100)/gi_{3}\right)}^{gi_{2}}}{1+{\left((t-100)/gi_{3}\right)}^{gi_{2}}},\;f_{2}^{100-220}(t)=ii_{1}\cdot \text{exp}\left(ii_{2}\cdot (t-100)\right)+ii_{3}$$

From 90 to 100 min, the values of *f*_1_ and *f*_2_ were fixed at 0 (mg dL^–1^ min^–1^ or μU dL^–1^ min^–1^). The parameters *gc*_*j*_, *gi*_*j*_, and *ii*_*j*_ (*j =* 1, 2, 3) are estimated to reproduce infusion rates for each subject with a nonlinear least squares technique [50].

We also considered alternative infusion patterns of *influx G* to *G* or *Y*, and *influx I* to *I* or *X* (Equations in S2 Table). Each pattern was fitted to the clamp data, and compared based on residual sum of the square (RSS) (see below) for each subject (S2 Table). The above model (Fig 1A), *influx G* to *Y* and *influx I* to *X*, appeared to be the best model.

### Parameter estimation

Blood glucose and insulin concentrations of each subject were normalized by dividing them by the respective maximum value of each time course. The model parameters for each subject were estimated to reproduce the normalized time course by a meta-evolutionary programming method to approach the neighborhood of the local minimum, followed by application of the nonlinear least squares technique to reach the local minimum [51]. For these methods, the parameters were estimated to minimize the objective function value, which is defined as residual sum of the square (RSS) between the actual time course obtained by clamp analyses and the model trajectories. RSS is given by the equation

$$RSS=\frac{n_{I}}{n_{G}+n_{I}}\sum_{i=1}^{n_{G}}{\left[G(t_{i})-G_{sim}(t_{i})\right]}^{2}+\frac{n_{G}}{n_{G}+n_{I}}\sum_{i=1}^{n_{I}}{\left[I(t_{i})-I_{sim}(t_{i})\right]}^{2}$$

where *n*_*G*_ and *n*_*I*_ are the total numbers of time points of measuring blood glucose and insulin, respectively, for the hyperglycemic and hyperinsulinemic-euglycemic clamps, *t*_*i*_ is the time of i-th time point, *G*(*t*) is the time-averaged blood glucose concentration within the time range (*t* – 5) min to *t* min with every 1-min interval, and *I*(*t*) is the blood insulin concentration at *t* min. *G*_*sim*_(*t*) and *I*_*sim*_(*t*) are simulated blood glucose and insulin concentrations, calculated in the same way as *G*(*t*) and *I*(*t*), respectively. The numbers of parents and generations in the meta-evolutionary programming were 400 and 4000, respectively. The model parameters for all subjects are shown in S7 Table.

### Determination of parameter outliers

The outliers of model parameters were detected by the adjusted outlyingness (AO) [52]. The cutoff value of AO was *Q*_3_ + 1.5*e* ^3*MC*^ ⋅ *IQR*, where *Q*_3_, *MC*, and *IQR* are the third quartile, medcouple, and interquartile range, respectively. The medcouple is a robust measure of skewness [53]. The number of directions was set at 7000. Subjects found to have outlier parameters (three NGT, two IGT and three T2DM subjects) were excluded from further study.

### Parameter sensitivity analysis

We defined the individual parameter sensitivity for each subject [*S*(*f*(*x*), *x*)] as follows:

$$S(f(x),x)=\frac{\partial \text{log}\;f(x)}{\partial \text{log}\;x}=\frac{x}{f(x)}\cdot \frac{\partial f(x)}{\partial x}$$

where *x* is the parameter value and *f*(*x*) is ISI or AUC_IRI10_. The differentiation is numerically approximated by central difference $\frac{\partial f(x)}{\partial x}\approx \frac{f(x+\Delta x)-f(x-\Delta x)}{2\Delta x}$, and *x* + Δ*x* and *x* – Δ*x* were set so as to be increased [*x* (1.1*x*)] or decreased [*x* (0.9*x*)] by 10%, respectively. Finally, we defined the parameter sensitivity by the median of the individual parameter sensitivity for all subjects.

We examined the parameter sensitivity for 70 parameters consisting of all rate constants, the products of each pair of rate constants, and the quotients of each two rate constants. For the products of each two rate constants, *x* = *k*_i_ ∙ *k*_j_, we configured the 10% increased *x* and 10% decreased *x* by $1.1x=\sqrt{1.1}k_{i}\cdot \sqrt{1.1}k_{j}$ and $0.9x=\sqrt{0.9}k_{i}\cdot \sqrt{0.9}k_{j}$, respectively, and we adopted a similar approach for the quotients of each two parameters. The higher the absolute value of parameter sensitivity, the larger the effect of the parameter on ISI or AUC_IRI10_.

### Fitting by power function

We hypothesized that a power function accounted for the relation between two correlated model parameters or clinical indices. We used the function *f*(*x*) = *a*·*x* ^*b*^, where *x* is the model parameter on the horizontal axis in Figs 3A and 4A. The parameters *a* and *b* were estimated to minimize the RSS,

$$RSS=\sum {\left(y-f(x)\right)}^{2}$$

where *y* is the model parameter or clinical index on the vertical axis in Figs 3A and 4A, with the use of a nonlinear least squares technique.

### Calculation of clinical indices

HOMA-β:

[360 × fasting serum immunoreactive insulin (F-IRI) (μU/mL)/fasting plasma glucose (FPG) (mg/dL) – 63]

HOMA-IR:

[F-IRI (μU/mL) × FPG (mg/dL)/405]

Insulinogenic index (I.I.):

Ratio of the increment of serum IRI to that of plasma glucose at 30 min after the onset of the OGTT

[ΔIRI_(0–30 min)_ (μU/mL)/ΔPG_(0–30 min)_ (mg/dL)]

AUC_IRI10–90_ [31]:

Incremental area under the insulin concentration curve from 10 to 90 min during the hyperglycemic clamp

AUC_IRI120/PG120_:

Ratio of the area under the insulin concentration curve from 0 to 120 min to that for plasma glucose from 0 to 120 min, without using the data measured at 90 min, in the OGTT

[AUC_IRI(0–120 min)_ (μU/mL)/AUC_PG(0–120 min)_ (mg/dL)]

Matsuda index [54]:

$$\left[10,000/\sqrt{\text{FPG}(\text{mg}/\text{dL})\times \text{F}\;-\;\text{IRI}\;(\mu \;\text{U}/\text{mL})\times \bar{G}\times \bar{I}}\;\right]$$

where $\bar{G}$ and $\bar{I}$ are mean PG and serum IRI concentrations during the OGTT, respectively.

Oral DI [55, 56]:

[AUC_IRI120/PG120_ × Matsuda index]

### Statistical analysis

Unless indicated otherwise, data are expressed as the median with first and third quartiles. Medians of clinical indices and parameter values were compared among NGT, IGT, and T2DM subjects with the use of the Steel-Dwass test [32], a statistical nonparametric test for multiple comparisons. A *P* value of <0.05 was considered statistically significant.

## Supporting Information

S1 Fig Time courses of blood glucose and insulin concentrations for each subject. The time courses of blood glucose and insulin concentrations for each subject in the simulation (blue curves) and in the clamp measurements (red circles) are shown. Subjects #1 to #50 are NGT (green background), #51 to #68 are IGT (red background), and #69 to #121 are T2DM (blue background). Subjects shown with a gray background (three NGT, two IGT, and three T2DM) were excluded from further analysis as outliers (see Materials and Methods). The residual sum of the square (RSS) between the time course for the clamp and the model trajectory is shown for each subject. The distribution of RSS for all subjects is shown in the bottom plot.
(PDF)

S2 Fig Parameters showing the highest correlation with clinical indices. Scatter plots for the indicated measured clinical indices versus the highest correlated model parameters (S4 Table) were constructed. Each circle indicates the values for an individual subject, with *r* indicating the correlation coefficient and the *P* values being for testing the hypothesis of no correlation. The parameter that shows the highest correlation with AUC_IRI10_ is *k*_5_/*k*_7_ (S4 Table), which seems reasonable given that this ratio is the normalized rate constant of insulin secretion divided by that of insulin clearance and corresponds to apparent insulin secretion. *k*_5_ itself also showed a high correlation with AUC_IRI10_ (S4 Table). Together, *k*_5_/*k*_7_ and *k*_5_ thus characterize insulin secretory capacity. The parameter that shows the highest correlation with ISI is *k*_4_∙*k*_7_ (S4 Table), which seems reasonable given that *k*_4_∙*k*_7_ is the product of the rate constants of insulin sensitivity and insulin clearance. *k*_4_ itself showed a high correlation with ISI (S4 Table). Together, *k*_4_∙*k*_7_ and *k*_4_ thus characterize insulin sensitivity. Importantly, the product (*k*_4_∙*k*_5_) of *k*_5_/*k*_7_ and *k*_4_∙*k*_7_, which show the highest correlations with insulin secretion and sensitivity, respectively, itself showed a high correlation with both measured and simulated clamp DI (Fig 3B). Taken together with the data in Fig 2B, this finding suggests that *k*_4_∙*k*_5_ is a functional parameter that represents clamp DI in the feedback model (Figs 3 and 4).
(PDF)

S3 Fig Scatter plots for MCR versus Clamp DI or 120-min PG. Each circle corresponds to the values for a single subject.
(PDF)

S4 Fig Insulin clearance estimated by use of both hyperglycemic and hyperinsulinemic-euglycemic clamps or either one of them. **P < 0.01 (Steel-Dwass test).
(PDF)

S1 Table Characteristics of the three groups of study subjects. Data are means ± SD. BMI, body mass index; FPG, fasting plasma glucose concentration; 2-h PG, 2-h plasma glucose level during the OGTT; F-IRI, fasting serum immunoreactive insulin concentration.
(DOC)

S2 Table Tested patterns of influxes and RSS. All four patterns differ according to which variables were increased by *influx G* and *influx I*. Each pattern was fitted to the clamp data of each subject, and individual RSS’s were calculated. *P* values were determined by paired t-tests, and RSS average of each pair of patterns was significantly different at a level alpha of 0.05. The best pattern was therefore pattern A, and used in this study.
(DOC)

S3 Table Parameter sensitivity analysis for insulin sensitivity and insulin secretion. Medians of the indicated parameters for all subjects were used for parameter sensitivity analysis with regard to ISI and AUC_IRI10_ (see Materials and Methods). The higher the absolute value of the parameter, the higher the sensitivity. *P* values relative to the median for the top-ranked parameter were determined by the two-sided Wilcoxon rank sum test. Parameter sensitivity of *k*_7_ (–2.28) and that of *k*_5_ (1.50) for AUC_IRI10_ are not significantly different (*P* = 0.460)
(DOC)

S4 Table Correlation between model parameters and measured clinical indices. Correlation coefficients (*r*) for the model parameters versus the indicated clinical indices are listed in descending order of absolute value (see Materials and Methods). *P* values are for testing the hypothesis of no correlation (corrected by the number of correlation calculation: multiplied by 1190). HOMA-β, homeostasis model assessment of β-cell function; HOMA-IR, homeostasis model assessment of insulin resistance; I.I., insulinogenic index.
(XLS)

S5 Table Correlation between the clinical Indices. Correlation coefficients (*r*) for the indicated clinical indices are listed in descending order of absolute value (see Materials and Methods). *P* values are for testing the hypothesis of no correlation (corrected by the number of correlation calculation: multiplied by 289).
(XLS)

S6 Table Experimental measurements for each subject. Subjects shown with a gray background (three NGT, two IGT, and three T2DM) were excluded from further analysis as outliers (see Materials and Methods).
(XLS)

S7 Table Estimated parameters for each subject. Subjects shown with a gray background (three NGT, two IGT, and three T2DM) were excluded from further analysis as outliers (see Materials and Methods)
(XLS)
