# Supplementary figures and images for: Glucose Homeostatic Law: Insulin Clearance Predicts the Progression of Glucose Intolerance in Humans
Source: PLoS One. 2015 Dec 1;10(12):e0143880. doi: 10.1371/journal.pone.0143880 (PMC4666631; doi:10.1371/journal.pone.0143880)

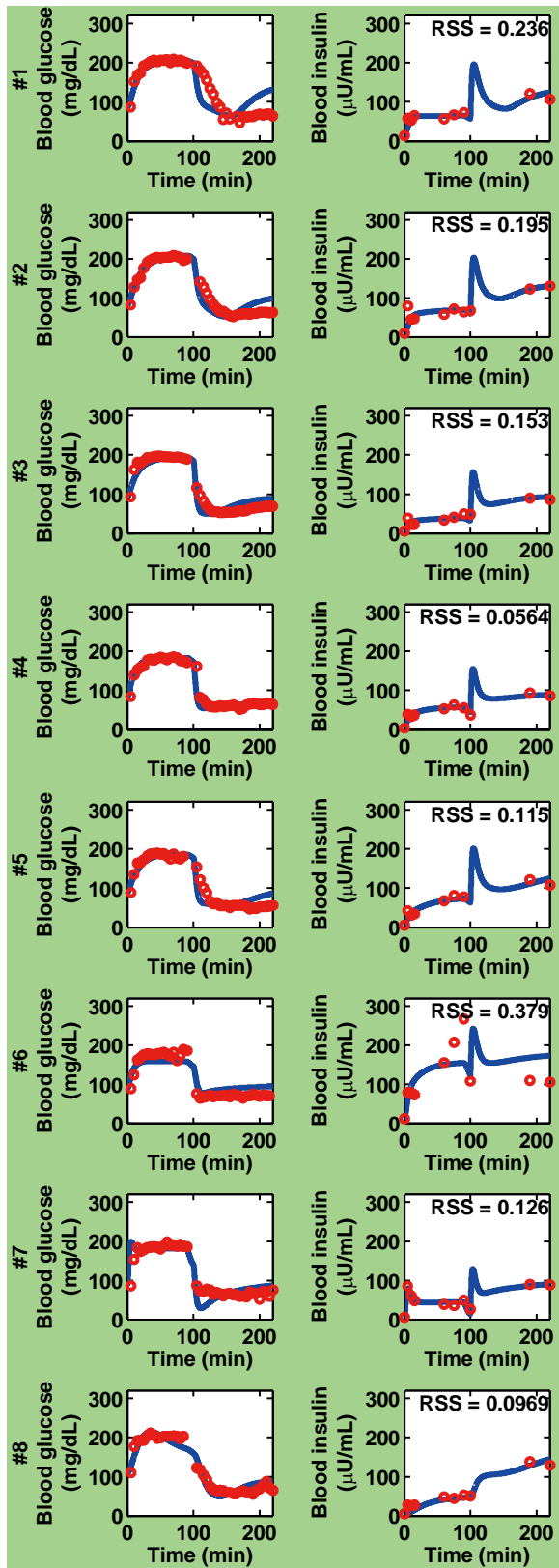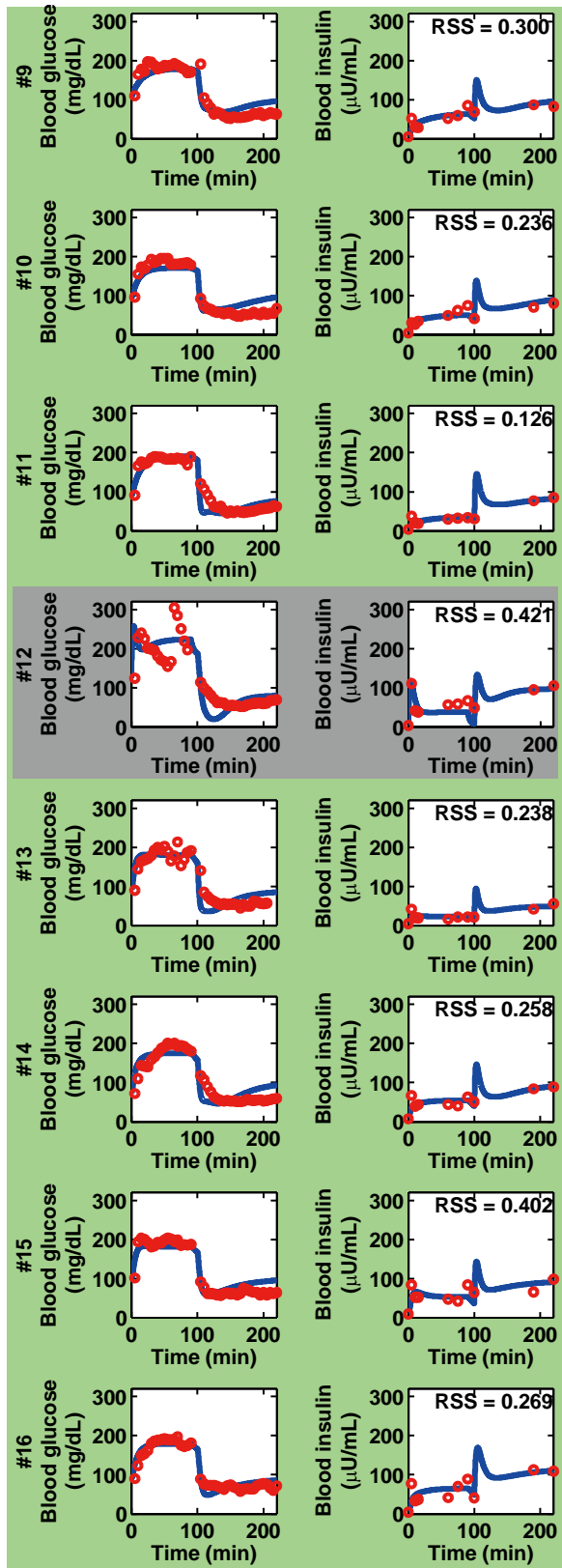

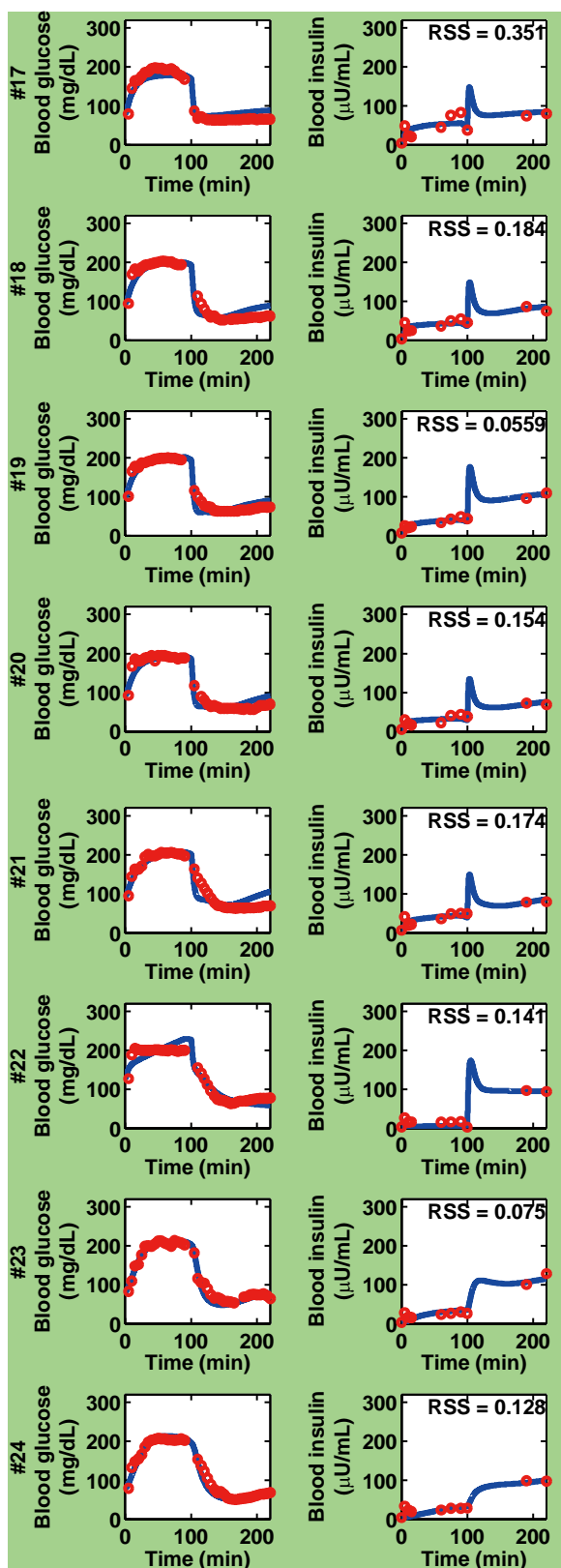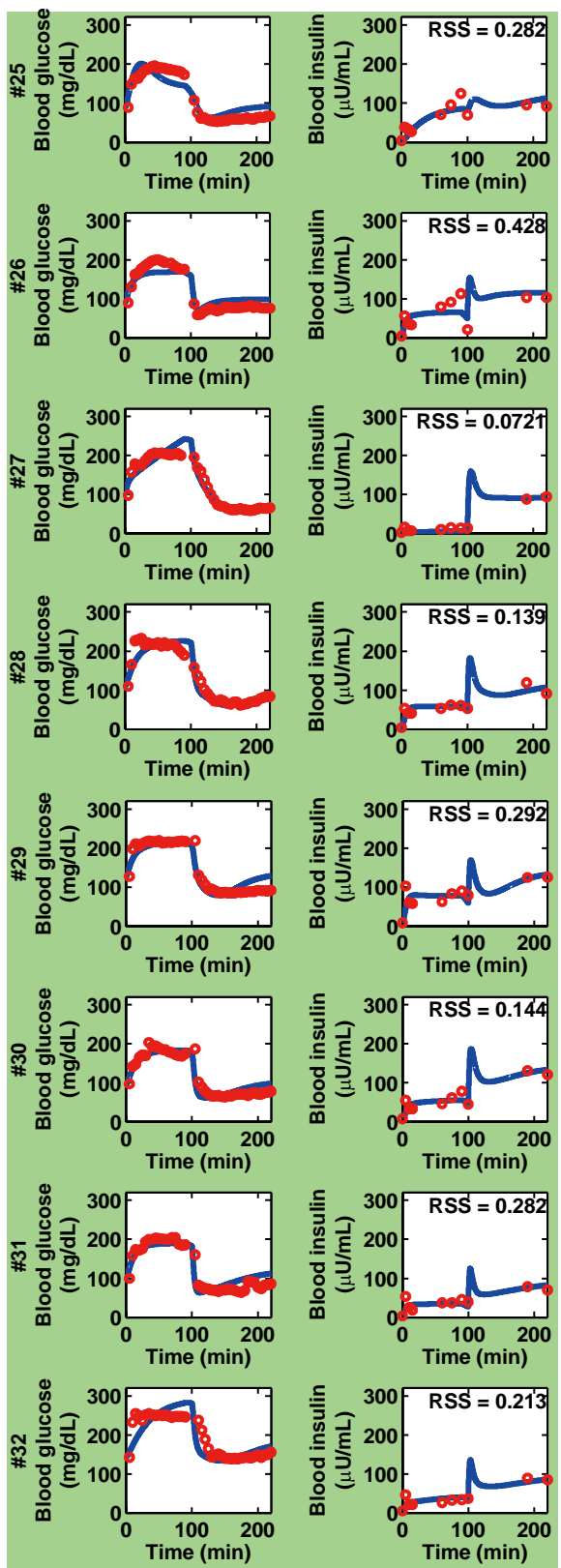

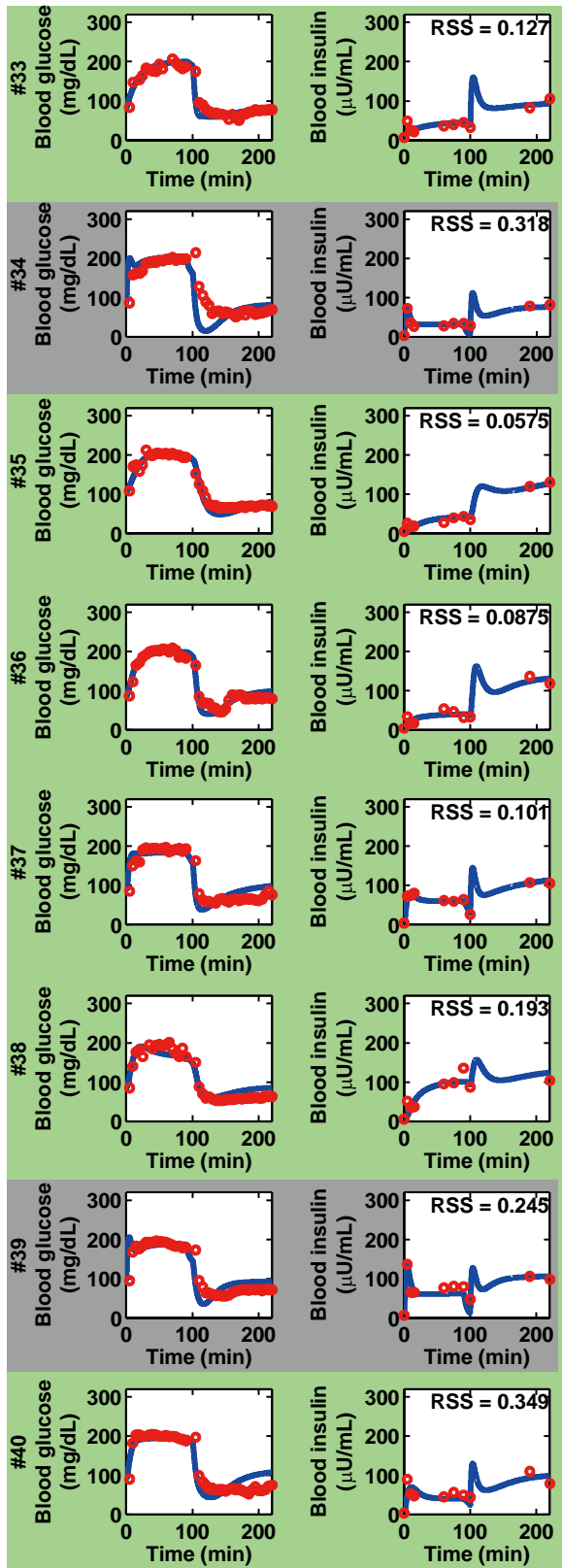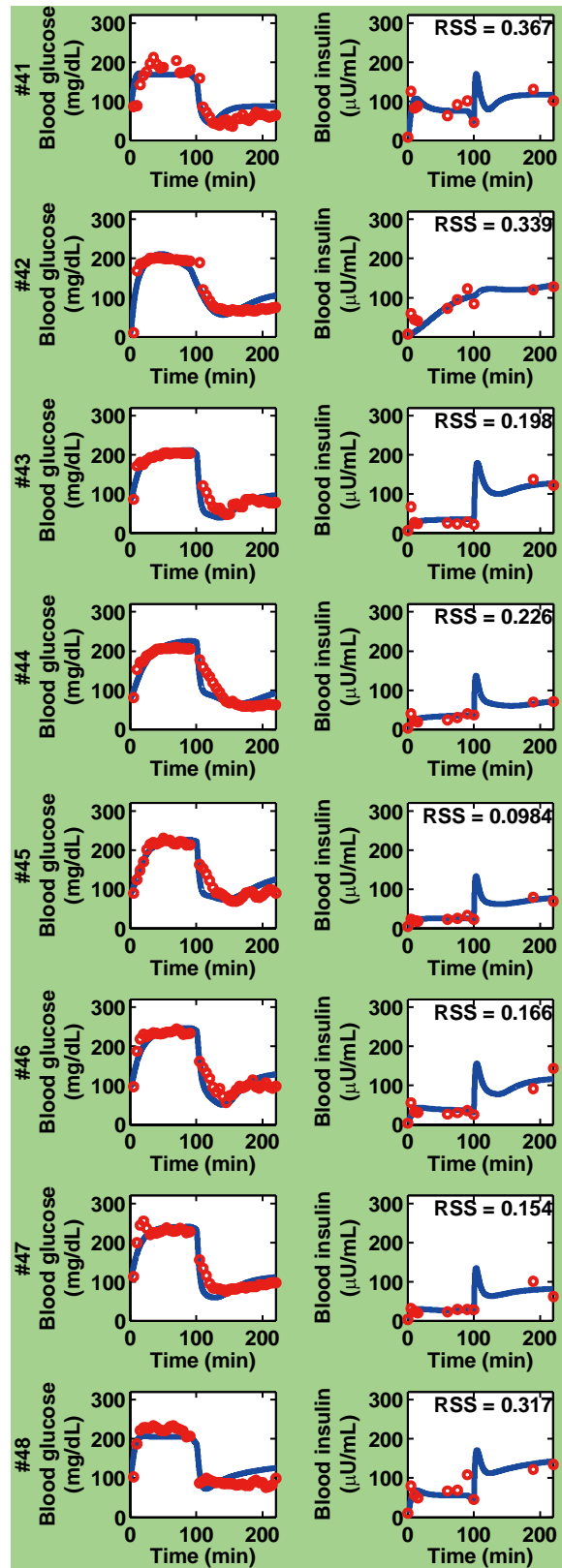

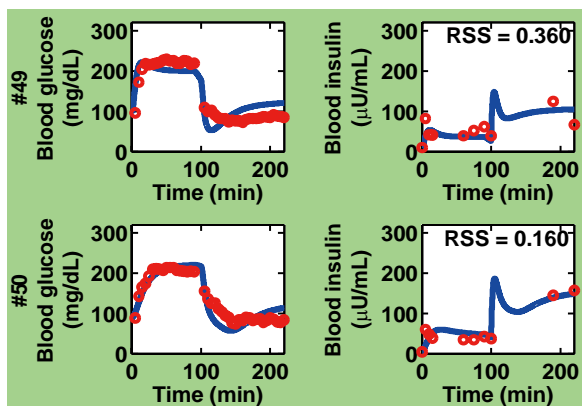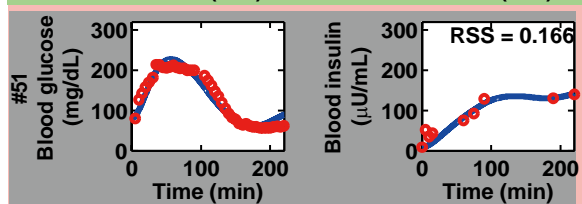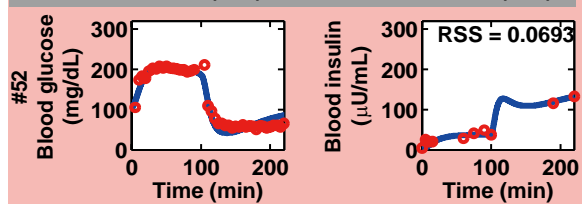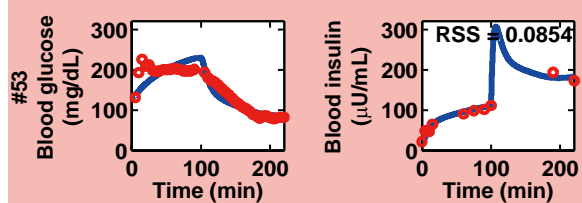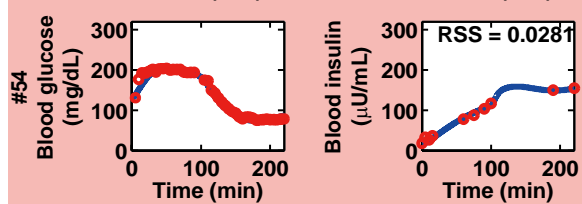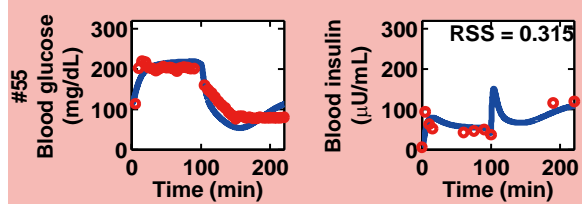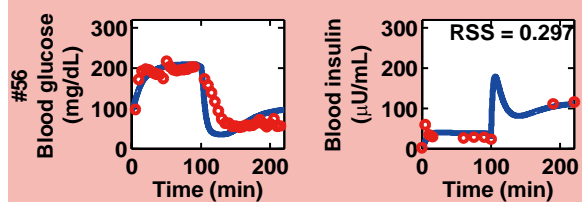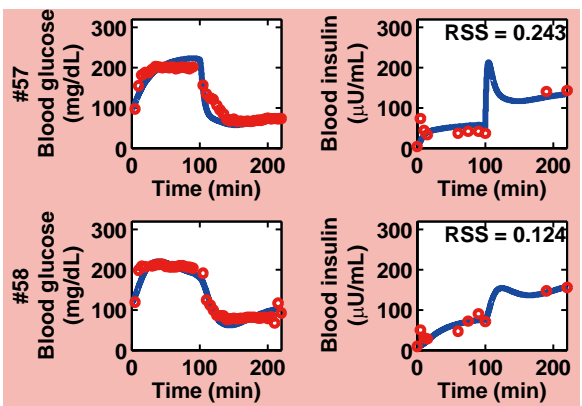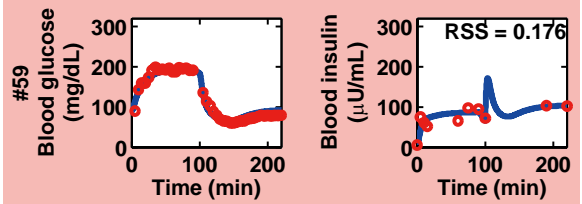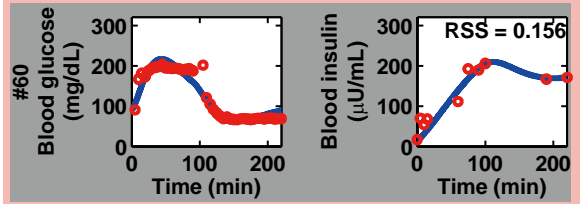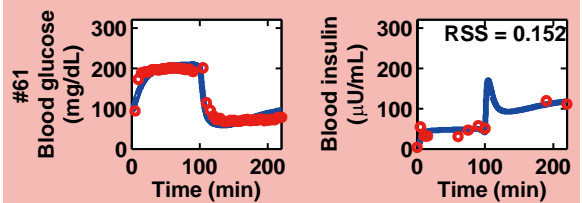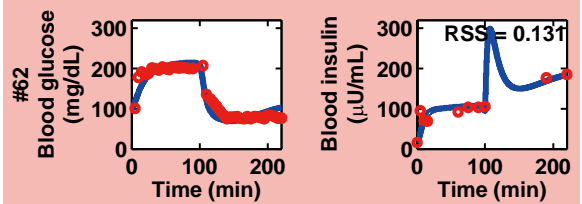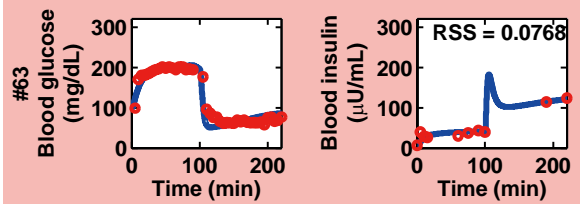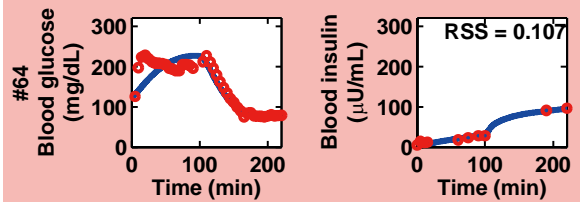

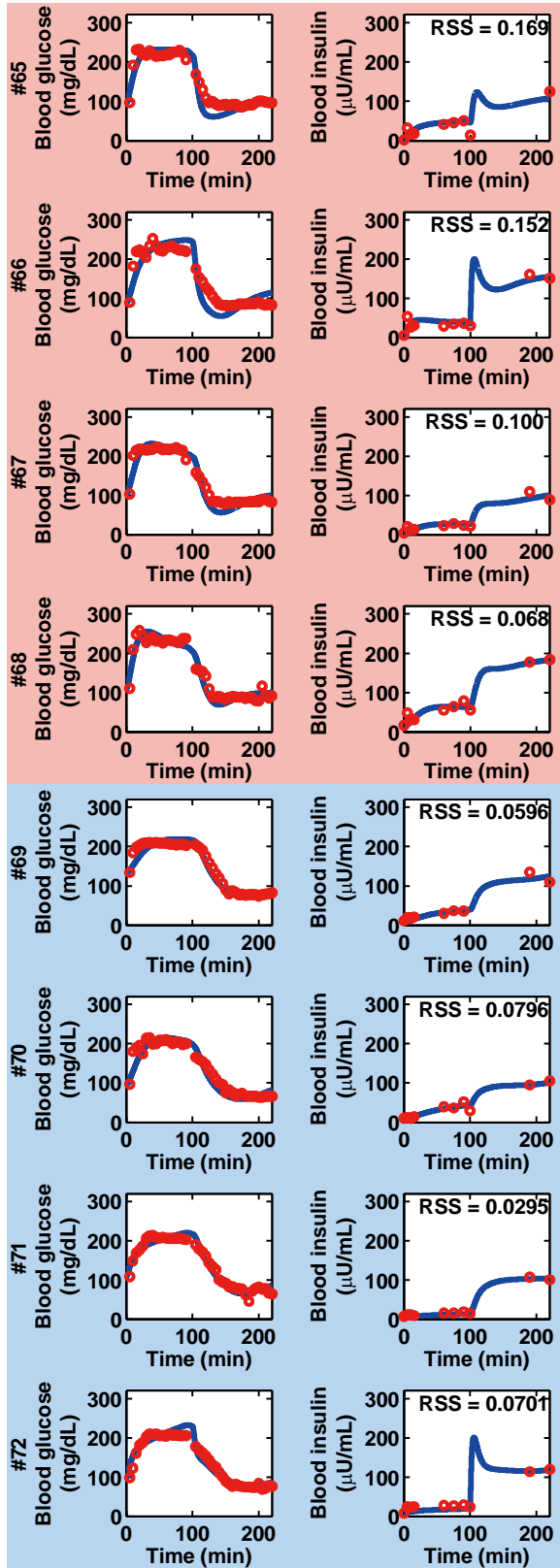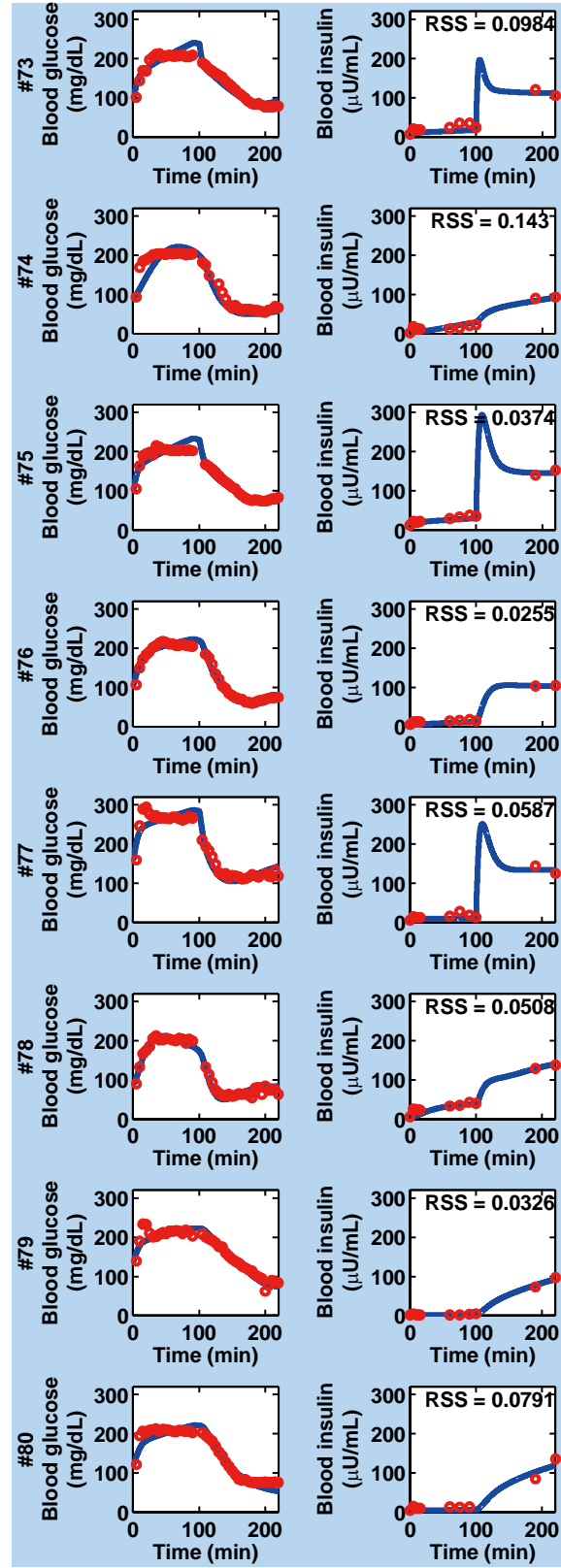

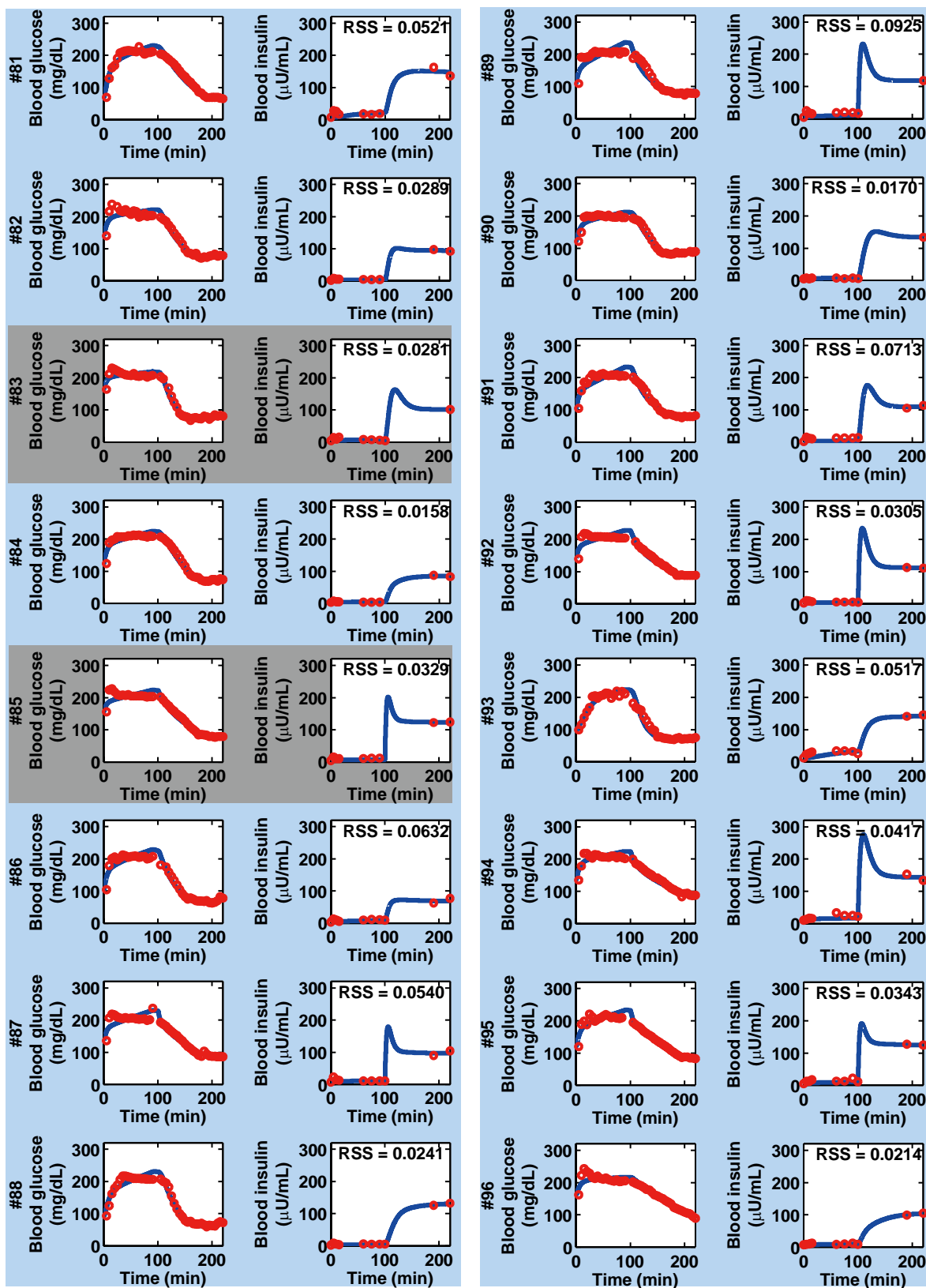

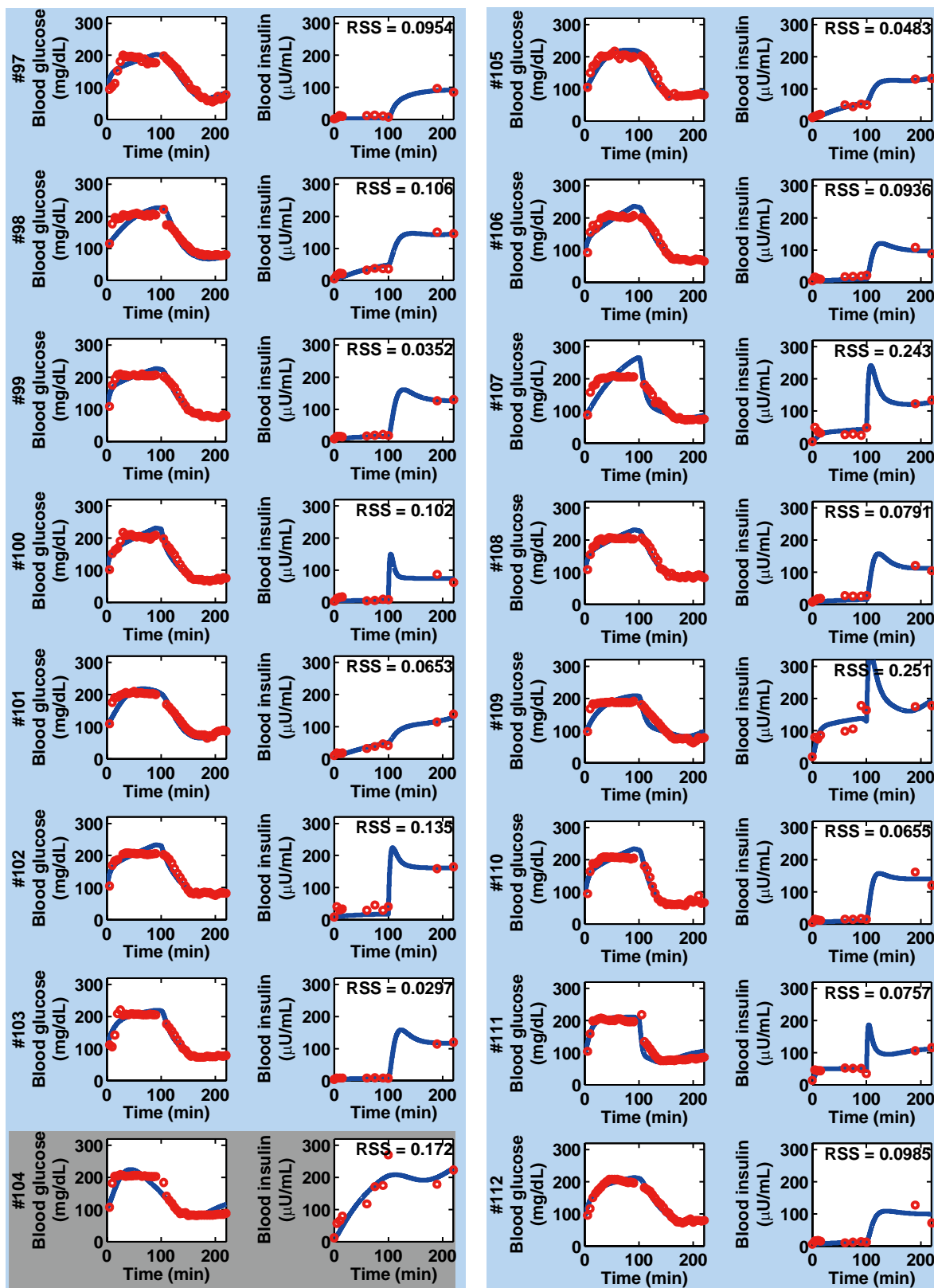

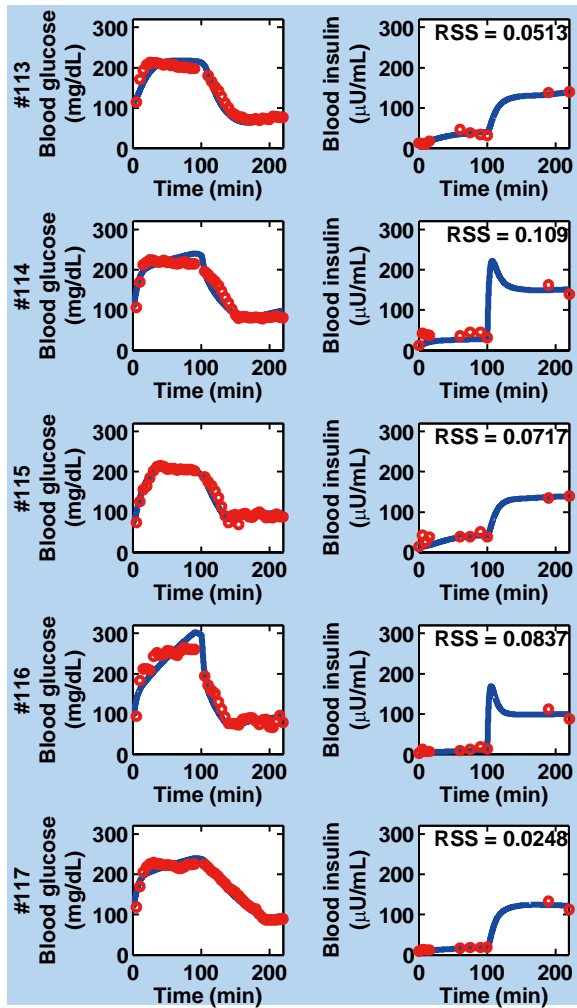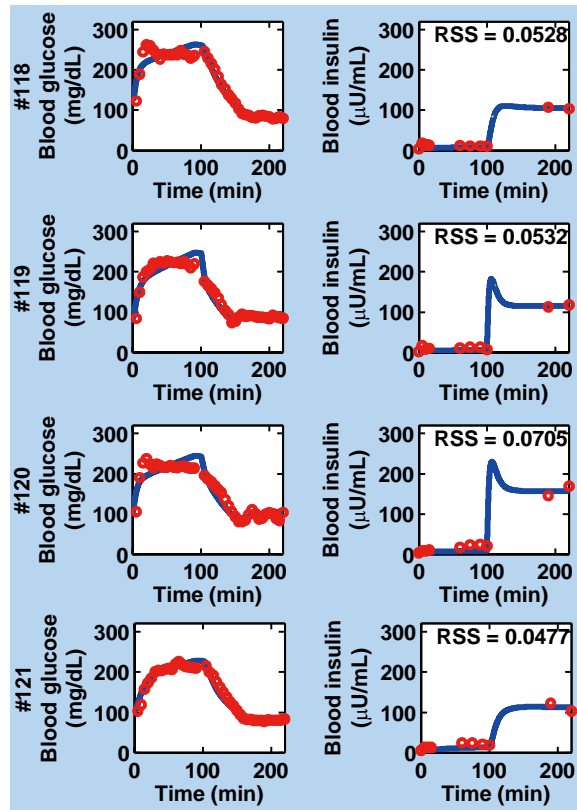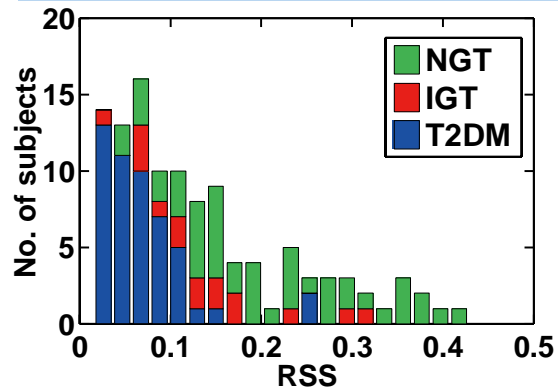

Supplement: S1 Fig — The time courses of blood glucose and insulin concentrations for each subject in the simulation (blue curves) and in the clamp measurements (red circles) are shown. Subjects #1 to #50 are NGT (green background), #51 to #68 are IGT (red background), and #69 to #121 are T2DM (blue background). Subjects shown with a gray background (three NGT, two IGT, and three T2DM) were excluded from further analysis as outliers (see Materials and Methods). The residual sum of the square (RSS) between the time course for the clamp and the model trajectory is shown for each subject. The distribution of RSS for all subjects is shown in the bottom plot. (PDF) [file pone.0143880.s001.pdf]

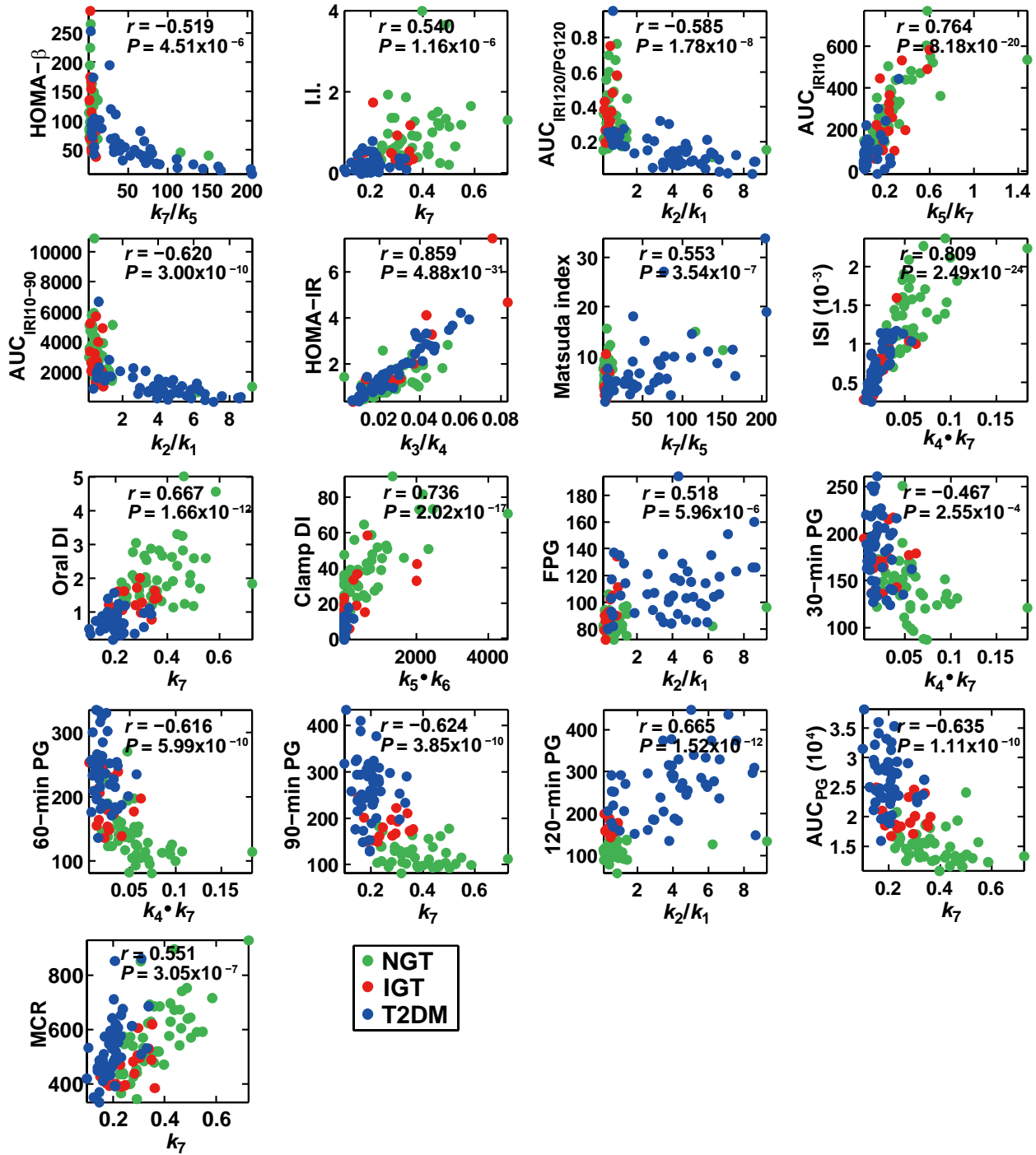

Supplement: S2 Fig — Scatter plots for the indicated measured clinical indices versus the highest correlated model parameters (S4 Table) were constructed. Each circle indicates the values for an individual subject, with r indicating the correlation coefficient and the P values being for testing the hypothesis of no correlation. The parameter that shows the highest correlation with AUCIRI10 is k 5/k 7 (S4 Table), which seems reasonable given that this ratio is the normalized rate constant of insulin secretion divided by that of insulin clearance and corresponds to apparent insulin secretion. k 5 itself also showed a high correlation with AUCIRI10 (S4 Table). Together, k 5/k 7 and k 5 thus characterize insulin secretory capacity. The parameter that shows the highest correlation with ISI is k 4∙k 7 (S4 Table), which seems reasonable given that k 4∙k 7 is the product of the rate constants of insulin sensitivity and insulin clearance. k 4 itself showed a high correlation with ISI (S4 Table). Together, k 4∙k 7 and k 4 thus characterize insulin sensitivity. Importantly, the product (k 4∙k 5) of k 5/k 7 and k 4∙k 7, which show the highest correlations with insulin secretion and sensitivity, respectively, itself showed a high correlation with both measured and simulated clamp DI (Fig 3B). Taken together with the data in Fig 2B, this finding suggests that k 4∙k 5 is a functional parameter that represents clamp DI in the feedback model (Figs 3 and 4). (PDF) [file pone.0143880.s002.pdf]

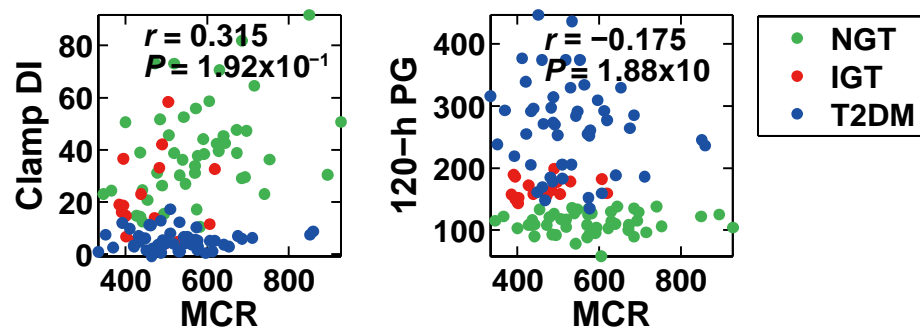

Supplement: S3 Fig — Each circle corresponds to the values for a single subject. (PDF) [file pone.0143880.s003.pdf]

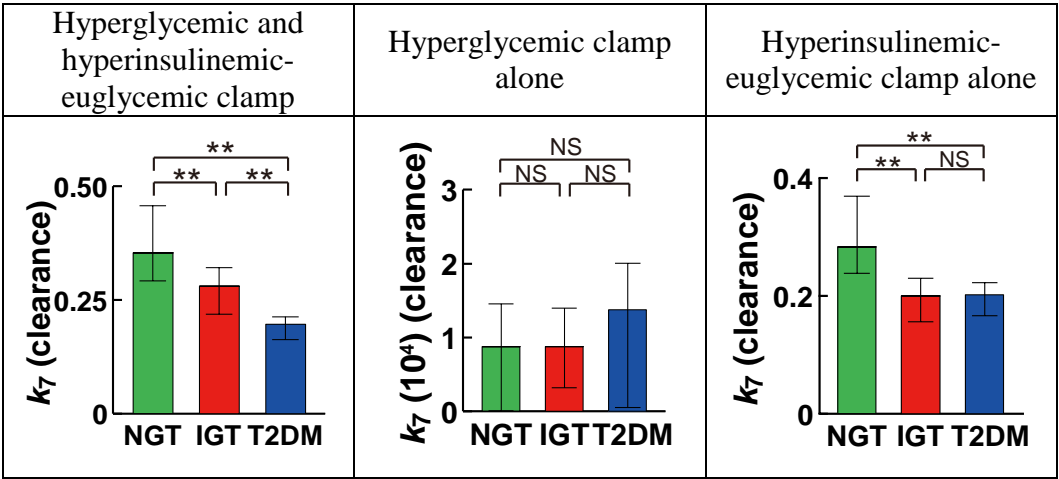

Supplement: S4 Fig — **P < 0.01 (Steel-Dwass test). (PDF) [file pone.0143880.s004.pdf]
