# Supplementary material for: Glucose Homeostatic Law: Insulin Clearance Predicts the Progression of Glucose Intolerance in Humans
Source: PLoS One. 2015 Dec 1;10(12):e0143880. doi: 10.1371/journal.pone.0143880 (PMC4666631; doi:10.1371/journal.pone.0143880)
Supplement: S1 Table — Data are means ± SD. BMI, body mass index; FPG, fasting plasma glucose concentration; 2-h PG, 2-h plasma glucose level during the OGTT; F-IRI, fasting serum immunoreactive insulin concentration. (DOC) [file pone.0143880.s005.doc]

|  | NGT | IGT | T2DM | Total |
| --- | --- | --- | --- | --- |
| Number | 47 | 16 | 50 | 113 |
| Sex (male/female) | 20/27 | 9/7 | 32/18 | 61/52 |
| Age (years) | 30.9 ± 8.74 | 42.9 ± 11.9 | 55.2 ± 14.1 | 43.2 ± 16.3 |
| BMI (kg/m^2^) | 21.1 ± 3.43 | 26.7 ± 6.89 | 26.0 ± 4.93 | 24.1 ± 5.31 |
| FPG (mg/dL) | 85.6 ± 6.70 | 93.0 ± 14.6 | 110 ± 22.4 | 97.5 ± 20.0 |
| 2-h PG (mg/dL) | 112 ± 17.3 | 167 ± 16.2 | 266 ± 74.2 | 187 ± 87.8 |
| F-IRI (μU/mL) | 5.65 ± 2.24 | 8.50 ± 5.56 | 6.80 ± 4.14 | 6.56 ± 3.82 |
