# Supplementary material for: Glucose Homeostatic Law: Insulin Clearance Predicts the Progression of Glucose Intolerance in Humans
Source: PLoS One. 2015 Dec 1;10(12):e0143880. doi: 10.1371/journal.pone.0143880 (PMC4666631; doi:10.1371/journal.pone.0143880)
Supplement: S2 Table — All four patterns differ according to which variables were increased by influx G and influx I. Each pattern was fitted to the clamp data of each subject, and individual RSS’s were calculated. P values were determined by paired t-tests, and RSS average of each pair of patterns was significantly different at a level alpha of 0.05. The best pattern was therefore pattern A, and used in this study. (DOC) [file pone.0143880.s006.doc]

| Pattern | Equation | | RSS Average ± SD |
| --- | --- | --- | --- |
| A | $\left\{\begin{matrix} \frac{dG}{dt}=flux 1-flux 2+flux 3-flux 4 \\ \frac{dI}{dt}=flux 6-flux 7 \\ \frac{dY}{dt}=-flux 1+flux 2+influx G \\ \frac{dX}{dt}=flux 5-flux 6+influx I \end{matrix}\right.$ | | 0.1395 ± 0.1037 |
| B | $\left\{\begin{matrix} \frac{dG}{dt}=flux 1-flux 2+flux 3-flux 4+influx G \\ \frac{dI}{dt}=flux 6-flux 7 \\ \frac{dY}{dt}=-flux 1+flux 2 \\ \frac{dX}{dt}=flux 5-flux 6+influx I \end{matrix}\right.$ | | 0.5132± 0.5784 |
| C | $\left\{\begin{matrix} \frac{dG}{dt}=flux 1-flux 2+flux 3-flux 4 \\ \frac{dI}{dt}=flux 6-flux 7+influx I \\ \frac{dY}{dt}=-flux 1+flux 2+influx G \\ \frac{dX}{dt}=flux 5-flux 6 \end{matrix}\right.$ | | 0.1457 ± 0.1003 |
| D | $\left\{\begin{matrix} \frac{dG}{dt}=flux 1-flux 2+flux 3-flux 4+influx G \\ \frac{dI}{dt}=flux 6-flux 7+influx I \\ \frac{dY}{dt}=-flux 1+flux 2 \\ \frac{dX}{dt}=flux 5-flux 6 \end{matrix}\right.$ | | 0.5159 ± 0.5764 |
|  |  |  |  |
| Test pair | *P* |  |  |
| A vs. B | 5.46×10^-13^ |  |  |
| A vs. C | 2.15×10^-8^ |  |  |
| A vs. D | 3.43×10^-13^ |  |  |
| B vs. C | 1.54×10^-12^ |  |  |
| B vs. D | 6.28×10^-5^ |  |  |
| C vs. D | 9.69×10^-13^ |  |  |
