# Supplementary material for: Glucose Homeostatic Law: Insulin Clearance Predicts the Progression of Glucose Intolerance in Humans
Source: PLoS One. 2015 Dec 1;10(12):e0143880. doi: 10.1371/journal.pone.0143880 (PMC4666631; doi:10.1371/journal.pone.0143880)
Supplement: S3 Table — Medians of the indicated parameters for all subjects were used for parameter sensitivity analysis with regard to ISI and AUCIRI10 (see Materials and Methods). The higher the absolute value of the parameter, the higher the sensitivity. P values relative to the median for the top-ranked parameter were determined by the two-sided Wilcoxon rank sum test. Parameter sensitivity of k 7 (–2.28) and that of k 5 (1.50) for AUCIRI10 are not significantly different (P = 0.460) (DOC) [file pone.0143880.s007.doc]

| Rank | ISI | | | AUC_IRI10_ | | |
| --- | --- | --- | --- | --- | --- | --- |
|  |  | Median | *P* |  | Median | *P* |
| 1 | *k*_4_ | 9.77×10^-1^ | 1.00 | *k*_7_ | –2.28 | 1.00 |
| 2 | *k*_1_/*k*_4_ | –5.29×10^-1^ | 1.40×10^-38^ | *k*_7_/*k*_5_ | –1.90 | 1.87×10^-1^ |
| 3 | *k*_4_/*k*_1_ | 5.26×10^-1^ | 1.40×10^-38^ | *k*_5_/*k*_7_ | 1.90 | 2.03×10^-1^ |
| 4 | *k*_3_/*k*_4_ | –5.08×10^-1^ | 1.40×10^-38^ | *k*_5_•*k*_6_ | 1.60 | 2.26×10^-3^ |
| 5 | *k*_4_/*k*_3_ | 5.06×10^-1^ | 1.40×10^-38^ | *k*_5_ | 1.50 | 4.60×10^-1^ |
| 6 | *k*_4_•*k*_7_ | 4.96×10^-1^ | 1.40×10^-38^ | *k*_6_/*k*_7_ | 1.36 | 2.02×10^-3^ |
| 7 | *k*_2_•*k*_4_ | 4.95×10^-1^ | 1.40×10^-38^ | *k*_7_/*k*_6_ | –1.35 | 2.09×10^-3^ |
| 8 | *k*_4_•*k*_6_ | 4.93×10^-1^ | 1.40×10^-38^ | *k*_1_•*k*_7_ | –1.27 | 5.51×10^-5^ |
| 9 | *k*_5_/*k*_4_ | –4.91×10^-1^ | 1.40×10^-38^ | *k*_7_/*k*_2_ | –1.24 | 3.40×10^-5^ |
| 10 | *k*_4_•*k*_5_ | 4.90×10^-1^ | 1.40×10^-38^ | *k*_2_/*k*_7_ | 1.24 | 3.43×10^-5^ |
| 11 | *k*_2_/*k*_4_ | –4.89×10^-1^ | 1.40×10^-38^ | *k*_7_/*k*_3_ | –1.14 | 2.15×10^-5^ |
| 12 | *k*_4_/*k*_5_ | 4.88×10^-1^ | 1.40×10^-38^ | *k*_4_•*k*_7_ | –1.14 | 2.17×10^-5^ |
| 13 | *k*_4_/*k*_2_ | 4.86×10^-1^ | 1.40×10^-38^ | *k*_3_•*k*_7_ | –1.14 | 2.11×10^-5^ |
| 14 | *k*_7_/*k*_4_ | –4.84×10^-1^ | 1.40×10^-38^ | *k*_7_/*k*_4_ | –1.14 | 2.11×10^-5^ |
| 15 | *k*_6_/*k*_4_ | –4.83×10^-1^ | 1.40×10^-38^ | *k*_3_/*k*_7_ | 1.14 | 2.15×10^-5^ |
| 16 | *k*_4_/*k*_7_ | 4.81×10^-1^ | 1.40×10^-38^ | *k*_4_/*k*_7_ | 1.14 | 2.11×10^-5^ |
| 17 | *k*_4_/*k*_6_ | 4.81×10^-1^ | 1.40×10^-38^ | *k*_2_•*k*_7_ | –9.47×10^–1^ | 9.99×10^-6^ |
| 18 | *k*_3_•*k*_4_ | 4.73×10^-1^ | 1.40×10^-38^ | *k*_1_/*k*_7_ | 9.06×10^–1^ | 7.74×10^-6^ |
| 19 | *k*_1_•*k*_4_ | 4.42×10^-1^ | 1.40×10^-38^ | *k*_7_/*k*_1_ | –9.03×10^–1^ | 7.45×10^-6^ |
| 20 | *k*_1_ | –8.79×10^-2^ | 1.40×10^-38^ | *k*_1_/*k*_5_ | –8.13×10^-1^ | 1.51×10^-8^ |
| 21 | *k*_1_•*k*_3_ | –6.16×10^-2^ | 1.40×10^-38^ | *k*_5_/*k*_1_ | 8.09×10^-1^ | 1.04×10^-8^ |
| 22 | *k*_6_/*k*_1_ | 5.97×10^-2^ | 1.40×10^-38^ | *k*_2_•*k*_5_ | 7.69×10^-1^ | 1.20×10^-10^ |
| 23 | *k*_1_/*k*_6_ | –5.97×10^-2^ | 1.40×10^-38^ | *k*_4_/*k*_5_ | –7.53×10^-1^ | 9.89×10^-12^ |
| 24 | *k*_1_/*k*_7_ | –5.51×10^-2^ | 1.40×10^-38^ | *k*_3_/*k*_5_ | –7.52×10^-1^ | 8.58×10^-12^ |
| 25 | *k*_7_/*k*_1_ | 5.33×10^-2^ | 1.40×10^-38^ | *k*_5_/*k*_4_ | 7.49×10^-1^ | 6.84×10^-12^ |
| 26 | *k*_1_/*k*_2_ | –4.91×10^-2^ | 1.40×10^-38^ | *k*_3_•*k*_5_ | 7.49×10^-1^ | 6.74×10^-12^ |
| 27 | *k*_2_/*k*_1_ | 4.88×10^-2^ | 1.40×10^-38^ | *k*_5_/*k*_3_ | 7.49×10^-1^ | 6.46×10^-12^ |
| 28 | *k*_1_/*k*_5_ | –4.74×10^-2^ | 1.40×10^-38^ | *k*_4_•*k*_5_ | 7.48×10^-1^ | 6.10×10^-12^ |
| 29 | *k*_5_/*k*_1_ | 4.71×10^-2^ | 1.40×10^-38^ | *k*_2_/*k*_5_ | –7.15×10^-1^ | 6.26×10^-13^ |
| 30 | *k*_1_•*k*_5_ | –3.54×10^-2^ | 1.40×10^-38^ | *k*_5_/*k*_2_ | 7.11×10^-1^ | 4.44×10^-13^ |
| 31 | *k*_1_•*k*_2_ | –3.43×10^-2^ | 1.40×10^-38^ | *k*_1_•*k*_5_ | 6.79×10^-1^ | 7.81×10^-14^ |
| 32 | *k*_3_ | –3.32×10^-2^ | 1.40×10^-38^ | *k*_6_/*k*_5_ | –5.69×10^-1^ | 2.82×10^-6^ |
| 33 | *k*_1_•*k*_6_ | –3.25×10^-2^ | 1.40×10^-38^ | *k*_5_/*k*_6_ | 5.66×10^-1^ | 2.60×10^-6^ |
| 34 | *k*_1_•*k*_7_ | –3.04×10^-2^ | 1.40×10^-38^ | *k*_6_•*k*_7_ | –4.50×10^-1^ | 1.66×10^-17^ |
| 35 | *k*_3_/*k*_7_ | –2.51×10^-2^ | 1.40×10^-38^ | *k*_6_/*k*_1_ | 3.05×10^-1^ | 5.30×10^-10^ |
| 36 | *k*_7_/*k*_3_ | 2.48×10^-2^ | 1.40×10^-38^ | *k*_1_/*k*_6_ | –3.05×10^-1^ | 5.37×10^-10^ |
| 37 | *k*_1_/*k*_3_ | –2.44×10^-2^ | 1.40×10^-38^ | *k*_2_•*k*_6_ | 2.78×10^-1^ | 4.36×10^-10^ |
| 38 | *k*_3_/*k*_1_ | 2.29×10^-2^ | 1.40×10^-38^ | *k*_1_ | –1.71×10^-1^ | 5.77×10^-35^ |
| 39 | *k*_3_/*k*_2_ | –1.90×10^-2^ | 1.40×10^-38^ | *k*_5_•*k*_7_ | 1.69×10^-1^ | 1.92×10^-12^ |
| 40 | *k*_2_/*k*_3_ | 1.90×10^-2^ | 1.40×10^-38^ | *k*_2_/*k*_1_ | 1.11×10^-1^ | 2.99×10^-35^ |
| 41 | *k*_5_/*k*_3_ | 1.85×10^-2^ | 1.40×10^-38^ | *k*_1_/*k*_2_ | –1.11×10^-1^ | 2.44×10^-35^ |
| 42 | *k*_3_/*k*_5_ | –1.84×10^-2^ | 1.40×10^-38^ | *k*_1_•*k*_4_ | –8.63×10^-2^ | 1.40×10^-38^ |

| Rank | ISI | | | AUC_IRI10_ | | |
| --- | --- | --- | --- | --- | --- | --- |
|  |  | Median | *P* |  | Median | *P* |
| 43 | *k*_6_/*k*_3_ | 1.84×10^-2^ | 1.40×10^-38^ | *k*_3_/*k*_1_ | 8.63×10^-2^ | 1.40×10^-38^ |
| 44 | *k*_3_/*k*_6_ | –1.84×10^-2^ | 1.40×10^-38^ | *k*_1_/*k*_3_ | –8.60×10^-2^ | 1.40×10^-38^ |
| 45 | *k*_6_•*k*_7_ | 1.57×10^-2^ | 1.40×10^-38^ | *k*_4_/*k*_1_ | 8.58×10^-2^ | 1.40×10^-38^ |
| 46 | *k*_3_•*k*_5_ | –1.54×10^-2^ | 1.40×10^-38^ | *k*_1_•*k*_3_ | –8.56×10^-2^ | 1.40×10^-38^ |
| 47 | *k*_7_ | 1.49×10^-2^ | 1.40×10^-38^ | *k*_1_/*k*_4_ | –8.53×10^-2^ | 1.40×10^-38^ |
| 48 | *k*_2_•*k*_3_ | –1.22×10^-2^ | 1.40×10^-38^ | *k*_2_ | 5.47×10^-2^ | 1.54×10^-35^ |
| 49 | *k*_3_•*k*_6_ | –1.20×10^-2^ | 1.40×10^-38^ | *k*_1_•*k*_6_ | –4.80×10^-2^ | 2.55×10^-10^ |
| 50 | *k*_2_•*k*_7_ | 9.26×10^-3^ | 1.40×10^-38^ | *k*_1_•*k*_2_ | –3.13×10^-2^ | 1.40×10^-38^ |
| 51 | *k*_5_•*k*_7_ | 7.79×10^-3^ | 1.40×10^-38^ | *k*_4_/*k*_2_ | –2.84×10^-2^ | 1.40×10^-38^ |
| 52 | *k*_3_•*k*_7_ | –7.18×10^-3^ | 1.40×10^-38^ | *k*_2_/*k*_4_ | 2.82×10^-2^ | 1.40×10^-38^ |
| 53 | *k*_5_/*k*_6_ | –6.73×10^-3^ | 1.40×10^-38^ | *k*_2_•*k*_3_ | 2.79×10^-2^ | 1.40×10^-38^ |
| 54 | *k*_6_/*k*_5_ | 6.68×10^-3^ | 1.40×10^-38^ | *k*_3_/*k*_2_ | –2.70×10^-2^ | 1.40×10^-38^ |
| 55 | *k*_2_ | 6.33×10^-3^ | 1.40×10^-38^ | *k*_2_/*k*_3_ | 2.69×10^-2^ | 1.40×10^-38^ |
| 56 | *k*_2_/*k*_7_ | –5.83×10^-3^ | 1.40×10^-38^ | *k*_2_•*k*_4_ | 2.65×10^-2^ | 1.40×10^-38^ |
| 57 | *k*_7_/*k*_2_ | 5.82×10^-3^ | 1.40×10^-38^ | *k*_6_/*k*_2_ | –1.09×10^-2^ | 3.03×10^-10^ |
| 58 | *k*_2_•*k*_6_ | 5.25×10^-3^ | 1.40×10^-38^ | *k*_2_/*k*_6_ | 1.09×10^-2^ | 3.19×10^-10^ |
| 59 | *k*_5_/*k*_7_ | –5.09×10^-3^ | 1.40×10^-38^ | *k*_6_/*k*_4_ | 5.88×10^-3^ | 3.78×10^-10^ |
| 60 | *k*_7_/*k*_5_ | 4.98×10^-3^ | 1.40×10^-38^ | *k*_4_/*k*_6_ | –5.88×10^-3^ | 3.83×10^-10^ |
| 61 | *k*_2_/*k*_5_ | 3.46×10^-3^ | 1.40×10^-38^ | *k*_3_•*k*_6_ | 4.28×10^-3^ | 3.78×10^-10^ |
| 62 | *k*_5_/*k*_2_ | –3.11×10^-3^ | 1.40×10^-38^ | *k*_4_ | –2.21×10^-3^ | 1.40×10^-38^ |
| 63 | *k*_6_/*k*_7_ | –2.22×10^-3^ | 1.40×10^-38^ | *k*_3_/*k*_4_ | 1.73×10^-3^ | 1.40×10^-38^ |
| 64 | *k*_7_/*k*_6_ | 1.94×10^-3^ | 1.40×10^-38^ | *k*_4_/*k*_3_ | –1.59×10^-3^ | 1.40×10^-38^ |
| 65 | *k*_2_•*k*_5_ | 1.94×10^-3^ | 1.40×10^-38^ | *k*_3_ | 1.04×10^-3^ | 1.40×10^-38^ |
| 66 | *k*_5_•*k*_6_ | 1.71×10^-3^ | 1.40×10^-38^ | *k*_3_•*k*_4_ | –3.63×10^-4^ | 1.17×10^-38^ |
| 67 | *k*_6_/*k*_2_ | –1.10×10^-3^ | 1.40×10^-38^ | *k*_6_ | 3.56×10^-4^ | 4.12×10^-7^ |
| 68 | *k*_2_/*k*_6_ | 1.05×10^-3^ | 1.40×10^-38^ | *k*_4_•*k*_6_ | –2.57×10^-4^ | 3.78×10^-10^ |
| 69 | *k*_5_ | 6.87×10^-4^ | 1.40×10^-38^ | *k*_3_/*k*_6_ | 0.00 | 3.82×10^-10^ |
| 70 | *k*_6_ | 0.00 | 4.34×10^-39^ | *k*_6_/*k*_3_ | 0.00 | 3.77×10^-10^ |
